# Importance, structure, cultivability, and resilience of the bacterial microbiota during infection of laboratory-grown *Haematococcus* spp. by the blastocladialean pathogen *Paraphysoderma sedebokerense*: evidence for a domesticated microbiota and its potential for biocontrol

**DOI:** 10.1093/femsec/fiaf011

**Published:** 2025-01-20

**Authors:** Jeanne Miebach, David Green, Martina Strittmatter, Claire Mallinger, Lucie Le Garrec, Qian Yi Zhang, Pierre Foucault, Caroline Kunz, Claire M M Gachon

**Affiliations:** Faculté des Sciences et Ingénierie, Sorbonne Université, UFR 927, 75005 Paris, France; MCAM (Molécules de Communication et Adaptation des Micro-organismes) UMR 7245 – Muséum National d'Histoire Naturelle, CNRS, 43 rue Buffon, 75005 Paris, France; Scottish Association for Marine Science, Oban PA37 1QA, United Kingdom; Scottish Association for Marine Science, Oban PA37 1QA, United Kingdom; MCAM (Molécules de Communication et Adaptation des Micro-organismes) UMR 7245 – Muséum National d'Histoire Naturelle, CNRS, 43 rue Buffon, 75005 Paris, France; MCAM (Molécules de Communication et Adaptation des Micro-organismes) UMR 7245 – Muséum National d'Histoire Naturelle, CNRS, 43 rue Buffon, 75005 Paris, France; Scottish Association for Marine Science, Oban PA37 1QA, United Kingdom; MCAM (Molécules de Communication et Adaptation des Micro-organismes) UMR 7245 – Muséum National d'Histoire Naturelle, CNRS, 43 rue Buffon, 75005 Paris, France; UMR7618 iEES-Paris, Sorbonne Université, 75005 Paris, France; Faculté des Sciences et Ingénierie, Sorbonne Université, UFR 927, 75005 Paris, France; MCAM (Molécules de Communication et Adaptation des Micro-organismes) UMR 7245 – Muséum National d'Histoire Naturelle, CNRS, 43 rue Buffon, 75005 Paris, France; MCAM (Molécules de Communication et Adaptation des Micro-organismes) UMR 7245 – Muséum National d'Histoire Naturelle, CNRS, 43 rue Buffon, 75005 Paris, France; Scottish Association for Marine Science, Oban PA37 1QA, United Kingdom

**Keywords:** green alga, Chlorophyta, fungal pathogen, microbiome, metagenomics, domestication

## Abstract

Industrial production of the unicellular green alga *Haematococcus lacustris* is compromised by outbreaks of the fungal pathogen *Paraphysoderma sedebokerense* (Blastocladiomycota). Here, using axenic algal and fungal cultures and antibiotic treatments, we show that the bacterial microbiota of *H. lacustris* is necessary for the infection by *P. sedebokerense* and that its modulation affects the outcome of the interaction. We combined metagenomics and laboratory cultivation to investigate the diversity of the bacterial microbiota associated to three *Haematococcus* species and monitor its change upon *P. sedebokerense* infection. We unveil three types of distinct, reduced bacterial communities, which likely correspond to keystone taxa in the natural *Haematococcus* spp. microbiota. Remarkably, the taxonomic composition and functionality of these communities remained stable during infection. The major bacterial taxa identified in this study have been cultivated by us or others, paving the way to developing synthetic communities to experimentally explore interactions within this tripartite system. We discuss our results in the light of emerging evidence concerning the structuring and domestication of plant and animal microbiota, thus providing novel experimental tools and a new conceptual framework necessary to enable the engineering of *Haematococcus* spp. microbiota toward the biocontrol of *P. sedebokerense*.

## Teaser

Infection of laboratory-grown *Haematococcus* spp. by the blastocladialean pathogen *Paraphysoderma sedebokerense* unveils evidence for a domesticated microbiota, the structure, resilience, and cultivability of which opens new perspectives for biocontrol.

## Introduction

The single-celled green alga *Haematococcus pluvialis* (Chlorophyceae), synonymized as *Haematococcus lacustris* (Nakada and Ota 2016), is cultivated industrially to produce astaxanthin, a carotenoid with powerful colouring and strong antioxidant capacity (Villaró et al. 2021). Based on the investigation of 73 European strains, Allewaert et al. (2015) described two related species, *Haematococcus rubicundus* and *Haematococcus rubens*, which could potentially also be utilized for industrial astaxanthin production. For large-scale production, *H. lacustris* can be cultivated in open raceway ponds or in enclosed photobioreactors (Qin et al. 2023). Its production can be severely compromised by the pathogen *Paraphysoderma sedebokerense* belonging to the Blastocladiomycota. Cultures in open ponds are especially vulnerable to diseases as they are directly exposed to the environment (Yu et al. 2022). Although closed photobioreactors seem to limit this source of contamination compared to open ponds, *P. sedebokerense* can still lead to culture collapses. Moreover, the high costs of the installation and difficult cleaning of closed photobioreactors make them less competitive for some *Haematococcus* spp. production (Qin et al. 2023).

Among *Haematococcus* spp. (hereafter referred to as *Haematococcus*) contaminants, *P. sedebokerense* is recognized to be the most problematic to mass cultures (Han et al. 2013). Indeed, *P. sedebokerense* infection of *H. lacustris* leads to a collapse of the cultures within a few days (Gutman et al. 2009). This fungus was first discovered and isolated in Israel from a *H. lacustris* culture by Hoffman et al. (2008) and then described as *P. sedebokerense* (gen. et sp. nov) (Gutman et al. 2009, James et al. 2012). It was then independently detected and isolated in *Haematococcus* cultures in Portugal (Strittmatter et al. 2015), South Korea (Hwang et al. 2019), and China (Lin et al. 2021). *Paraphysoderma sedebokerense* was also shown to infect microalgae such as *Scenedesmus dimorphus* in the USA (Letcher et al. 2016) and *Chromochloris zofingiensis* (Alors et al. 2021). The highest infection prevalence, however, was found for *H. lacustris* cultivation (Gutman et al. 2009). Numerous strategies have been investigated to limit the blastocladialean contamination and maintain astaxanthin production; among them, acidic culture conditions (Hwang et al. 2019), use of surfactants (Ding et al. 2020), glufosinate application (Alors et al. 2022), and H_2_O_2_ treatments (Carney and Sorensen 2016) have been shown to have some efficacy. However, the complex and poorly understood life cycle of the fungus (Strittmatter et al. 2015), and the thick-walled cyst of *P. sedebokerense* that can withstand disinfection (Alors et al. 2023), both make control of this disease challenging.

Today, plant disease management is an enormous challenge to the agri industry, which increasingly is turning to biological control as one of the more environmentally benign alternatives to more classic pest control strategies. Exploiting microbial antagonisms, such as the production of antimicrobial compounds or the induction of plant resistance all emerge as possible biocontrol strategies (Syed Ab Rahman et al. 2018), its general aim is to engineer or breed individual microorganisms or entire microbial consortia that harbour beneficial microbes. One way to find such bacteria is to explore directly the microbiota of a given cultivated plant of interest. Liu et al. (2020) showed that recruitment of a dominant bacterial symbiont by wheat was able to mediate resistance to *Fusarium pseudograminearum*. Similarly Li et al. (2019) identified bacteria potentially involved in resistance mechanisms against the crown gall disease agent by comparing the endophytic bacterial communities of resistant and susceptible peach cultivars. Similarly, biocontrol using biological agents is of growing interest for microalgae as their commercial production increases worldwide (Carney and Lane 2014). For example, zooplankton such as *Daphnia* can prey on fungal spores, particularly chytrid zoospores, and can be an effective control (Kagami et al. 2004, Carney and Lane 2014). Fisher et al. (2019) highlighted a microbiome protective effect in microalgae cultures. Indeed, they showed that specific algal–bacterial cocultures with *Microchloropsis salina* protected cultures from rotifer grazing. In the case of *Haematococcus*, industrial cultivation setups in closed photobioreactors allow a controllable system in which microbiome engineering could be envisioned, and allow testing for biocontrol activity of specific microbiota to protect the algal cultures. To the best of our knowledge, no study has investigated the potential of the bacterial microbiota for the biocontrol of algal pathogens.

Algae, like plants, live in association with bacteria (Dittami et al. 2014, Seymour et al. 2017). In analogy to the rhizosphere, the term phycosphere has been postulated for four decades and describes the region (diffusive boundary layer) surrounding algal cells rich in nutrients and biologically active compounds, hosting key interactions ranging from mutualistic to algicidal (Seymour et al. 2017). Numerous effects of the algal microbiome on industrial production of microalgae have been investigated, such as the production of vitamins, fixation of nitrogen, or the liberation of CO_2_ and minerals (Dittami et al. 2014, Lian et al. 2018). Pathogens such as bacteria, fungi, zooplankton, or other microalgae can be part of the phycosphere or can be external contaminants that may cause destruction of industrial algal cultures (Carney and Sorensen 2016, Ding et al. 2020, Yu et al. 2022). In a recent study, Blifernez-Klassen et al. (2021) applying phylogenetic and functional analysis of the *Botryococcus* consortia, identified that the microalga maintains its own mutualistic microbial community that controls its surrounding biosphere including parasitic bacteria. Their study shows that *Botryococcus* niches auxotrophic bacteria such as *Brevundimonas* spp. depend on the microalga for their supply of biotin and in turn produce bacteriocin antibiotics that control the growth of the devastating pathogen, *Mycobacterium*. Other studies highlight that bioactive metabolites such as antibiotic compounds can be secreted by algae-associated bacteria and act as chemical defences (Abdul Malik et al. 2020, Krohn et al. 2022). Moreover, in a multiomics approach looking into microalga and bacteria interactions, it was shown that effector molecules known from plant–microbe interactions act as inducers of the innate immunity, and seem to be relevant at the evolutionary early plant–microbiome level (Krohn-Molt et al. 2017).

The few studies investigating the bacterial community associated to *Haematococcus* have mainly focussed on enhancing algal biomass or astaxanthin production. Kublanovskaya et al. (2020), described the natural microbial community associated with *H. lacustris* collected at different locations along the White Sea temporal rock ponds, revealing a total of 13 main bacterial phyla, but dominated by the Proteobacteria, Bacteroidetes, Firmicutes, and Cyanobacteria. When comparing these environmental algal isolates with their respective isolates grown under laboratory conditions, the diversity of the laboratory-grown strains was much reduced with only two phyla present: Proteobacteria and Actinobacteria. Bacteria from *Hydrogenophaga* genus were the only one present in both environmental and laboratory cultured *H. lacustris*. To enhance algal growth Lee et al. (2019) cocultured *Haematococcus* spp. with the bacteria *Achromobacter*, which produces the plant growth hormone indole-3-acetic acid. Lee et al. (2022) inoculated axenic *Haematococcus* spp. first with *Paenarthrobacter ureafaciens* then followed by *Sphingomonas hankookensis* that had been isolated from the algal microbiota, and observed a 2.1-fold increase in algal biomass. This study shows a successful example of engineering microbiota with key beneficial bacteria. Other studies focused on the shift in the bacterial microbiota under different stress conditions (oxidation, high light, and low nutrients). Among them, a patent (number US9113607B1) described a shift in the bacterial community of *H. lacustris* when applying H_2_O_2_ disinfection treatment in an industrial set-up (Carney and Sorensen 2016). Chekanov et al. (2021) characterized the dynamics of *H. lacustris* microbiota isolated from the White Sea coast and cultivated in a photobioreactor during astaxanthin accumulation. Proteobacteria and Bacteroidetes were the two dominant phyla, with *Caulobacter* bacteria becoming more abundant upon astaxanthin accumulation.

To the best of our knowledge, only one group has explored the effect of parasitism on the microbiota of microalgae: Hoeger et al. (2021, 2022) investigated the microbiota dynamics of four freshwater microalgae *Scenedesmus vacuolatus, Desmodesmus quadricauda, Chlorella sorokiniana*, and *Botryococcus braunii* when challenged by the endoparasite *Amoeboaphelidium protococcarum*. Upon infection by the aphelid, the bacterial diversity increased and the bacterial functional traits shifted to detoxification, degradation, and cellulolysis Hoeger et al. (2021, 2022). In higher plants, metagenomics have shown that *Fusarium* wilt disease induces organ-specific changes in the bacterial and fungal communities from chili peppers. Several functional genes involved in detoxification, biofilm formation, and plant microbiome signalling pathways, were enriched in the microbiome of diseased chili peppers (Gao et al. 2021). Recently, Russ et al. (2024) identified the microbiota associated with *Rhizoctonia solani* disease suppression in sugar beet seedlings after amending soil with a keratin-rich waste stream. Enrichment of bacterial families known in disease suppressive soil was observed, as well as an abundance of genes encoding for keratinolytic enzymes in the keratin-amended samples (Russ et al. 2024).

Here, we first show that bacteria from the *Haematococcus* microbiota affect the outcome of the infection by *P. sedebokerense*. We, thus set out to explore the tripartite ‘algae–microbiota–pathogen’ consortium to understand the role of the bacteria in the infection. Our hypothesis was that the microbiota of *Haematococcus* would be affected in composition and function following infection by *P. sedebokerense*, and such variations might be informative to identify bacterial taxa and biological functions directly relevant to the differences of resistance observed between different *Haematococcus* strains (Allewaert et al. 2018). By combining a metagenomic and a laboratory-based approach, we thus characterize the microbiota consortium upon pathogen infection in laboratory cultures, both taxonomically and functionally. Altogether this work proposes tools and insights to explore the potential of *Haematococcus* bacteria for biocontrol.

## Materials and methods

### Biological material

#### *Haematococcus* spp. cultures

The 44 clonal *Haematococcus* strains used in this study (Table S1) were obtained from the Culture Collection of Algae and Protozoa (CCAP) and Allewaert et al. (2015, 2018). For long-term maintenance, algal strains were kept on solid 3N-BBM+V agar medium (Bold Basal Medium with 3-fold nitrogen and vitamins, CCAP, www.ccap.ac.uk), at 15°C, with a light intensity of 80 μmol photons m^−2^ s^−1^, and a 12 h:12 h light–dark period. For routine maintenance, the algal strains were kept liquid 3N-BBM+V at 20°C with a light intensity of 25 μmol photons m^−2^ s^−1^, and a 12 h:12 h light–dark period and subcultured every 3 months.

To first assess the influence of antibiotics on the infection by *P. sedebokerense*, the *Haematococcus* strain Haemc1 (Table S1) was grown in the presence of a mix of ampicillin and kanamycin at a concentration of 3.75 mg l^−1^ each, or ampicillin alone at a concentration of 150 mg l^−1^ in duplicates. Infection rate was assessed by microscopy via counting of infected and uninfected *Haematococcus* cells at 8 and 29 days postinoculation (dpi). Samples were randomized and counted in independent duplicates.

#### *Paraphysoderma sedebokerense* cultures

Two axenic strains of the fungal pathogen *P. sedebokerense*, PS1 and FD61 (Letcher et al. 2016, Strittmatter et al. 2015, 2020) were used. Fungal cultures were maintained in 40 ml suspension culture flasks in liquid Chytrid Growth Medium (CGM, CCAP, www.ccap.ac.uk), at 25°C, light intensity of 80 μmol photons m^−2^ s^−1^, 12 h:12 h light–dark period and subcultured every 3–4 weeks.

#### Axenization of algal cultures

Axenization of *Haematococcus* strains was performed as follows: 3-week-old liquid algal cultures were treated with chloramphenicol 0.075 mg ml^−1^ in liquid 3N-BBM+V medium for 2 days, before being plated on 3N-BBM+V agar supplemented with the same chloramphenicol concentration. Bacteria-free algal colonies were picked using an inverted microscope in sterile conditions and transferred onto antibiotic-free CGM to check for absence of bacteria. After a few days of incubation, candidate bacteria-free algal colonies were transferred into liquid 3N-BBM+V. The axenic state of the *Haematococcus* strains was confirmed using 16S rRNA gene amplification (Fig. S1): 1 ml of candidate axenic *Haematococcus* culture was centrifuged, the supernatant removed, and DNA extraction followed by 16S rRNA gene amplification was carried out (see the section ‘Isolation and identification of cultivable bacteria’ for the detailed protocols). A nonaxenic *Haematococcus* culture was subjected to the same process as a positive control. *Pseudomonas* sp. DNA (positive control) and PCR mix plus MilliQ water (a negative control) were included for the Polymerase Chain Reaction (PCR). Additionally, axenicity of the algal cultures was confirmed by plating the candidate axenic algal cultures on Luria–Bertani (LB) growth medium. A nonaxenic *Haematococcus* culture was always subjected to the same process as a positive control.

### Isolation and identification of cultivable bacteria

The 44 *Haematococcus* strains kept on agar slants were subcultured in liquid 3N-BBM+V. For each of the 44 *Haematococcus* strains, serial dilutions in 3N-BBM+V were prepared (10^−2^, 10^−3^ and 10^− 4^). 10 μl of the dilutions were plated onto solid microbiota growth medium, made from 3N-BBM+V supplied with 20 g l^−1^ of peptone, 30 g l^−1^ of glucose, and 15 g l^−1^ of agar. Additionally, serial dilutions for some *Haematococcus* strains (BE03_05, CCAP 34/14, CZ01_06, IT01_09, and NL01_04 et SAG 192.80) were also plated onto solid LB medium. All agar plates were incubated at 25°C until bacterial colonies were visible (which corresponded to ~2 weeks depending on the bacterial isolates). Each visually identified morphotype was cultured in liquid medium and was stored at −80°C in 25% glycerol. Identification of the different bacterial morphotypes was accomplished either by directly suspending a bacterial colony into 20 μl of MilliQ water, which was incubated for 10 min at 80°C and directly used for PCR. When this method failed, DNA was first extracted using, the Quick-DNA Fungal/Bacterial Microprep Kit (Zymo Research). 16S rRNA gene amplification was done using Eubac27F (5′-AGAGTTTGATCCTGGCTCAG-3′) and 1492R (5′-GGTTACCTTGTTACGACT-3′) primers. The PCR reaction mix contained 1 μl of template DNA, 0.8 μl of dNTP (2.5 mmol l^−1^), 0.5 μl for each primer (10 ml), 1 μl of 10X buffer, and 0.05 μl DreamTaq polymerase (5 U μl**^−^**^1^). The mixture was adjusted to a final volume of 10 μl with MilliQ water. The PCR conditions on the thermocycler (TECHNE Prime) were as follows: 25 cycles (30 s at 95°C, 30 s at 51.2°C, and 1 min at 72°C) with an initial denaturing step of 3 min at 95°C and a final elongation step of 5 min at 72°C. The PCR products were checked by agarose gel electrophoresis and Sanger sequencing.

### Preparation of *P. sedebokerense* inoculum

The fungal inoculum was prepared as follows. *Paraphysoderma sedebokerense* cultures (strain PS1 or FD61) in CGM were centrifuged 5 min at 4000 r m^−1^, the supernatant was removed, and the pellet was washed with 3N-BBM+V and resuspended in 3N-BBM+V. This fungal suspension as such was used for the metagenomics approach. For all the other experiments, the fungal suspension was sequentially filtered through 25 μm and 10 μm pore size cloths to eliminate aggregated cysts and keep only cells smaller than 10 μm like amoebae.

### Inoculation of axenic and nonaxenic *Haematococcus* cultures and addition of synthetic communities

In a first attempt to rebuild a synthetic community (SynCom 1) resembling the microbiota of SAG 192.8, 10 clonal bacterial strains isolated from the *H. pluvialis* strains SAG 192.8, and belonging to the genera *Mesorhizobium, Aeromicrobium, Microbacterium, Brevundimonas*, and *Variovorax* were used. Bacteria cryoconserved in liquid LB with 25% glycerol at −80°C, were resuspended into liquid LB media without agitation. Once bacteria had grown in liquid LB, the suspension was plated on LB agar, incubated at 25°C and isolated colonies were then picked and resuspended in 3 ml liquid LB. This bacterial preculture was incubated under agitation overnight at 37°C for all bacterial genera except for *Variovorax* culture, which was incubated at 25°C. OD was measured at 600 nm and volumes corresponding to 0.3 OD for each bacterial culture were mixed together (Syncom 1), centrifuged at 13 000 r m^−1^ and washed two times with sterile 3N-BBM+V. 30 μl of Syncom 1 was added to 1.5 ml of axenic and nonaxenic *H. lacustris* SAG192.8 at a cell concentration of ∼ 2 × 10^5^ cells ml^−1^ and 150 μl of the fungal filtrate described above (∼2 × 10^5^ cells ml^−1^). In the second experiment, which was repeated twice independently with similar results, the synthetic community (SynCom 2) was reduced to nonredundant genera, and thus comprised of only five clonal bacterial strains, representing *Mesorhizobium, Aeromicrobium, Microbacterium, Brevundimonas*, and *Variovorax*. The preparation of Syncom 2 was identical to Syncom 1. Axenic and nonaxenic *H. rubicundus* IT01_06 and *H. lacustris* CCAP34/14 were inoculated following the same protocol as for SAG192.8. All samples were incubated at 23°C, with a light intensity of 50 μmol photons m^−2^ s^−1^, and a 12 h:12 h light–dark photo period. The qualitative rating of symptoms was performed by two experienced researchers following a double-blind procedure.

### Metagenomics analysis

#### Experimental setup

The metagenomics approach was split in two complementary parts (Table S1 and Fig. S2). To analyze the changes of the microbiota associated to individual *Haematococcus* strains, two *H. lacustris* and four *H. rubicundus* strains with contrasting levels of resistance to PS1 (Allewaert et al. 2018) were inoculated with *P. sedebokerense* (Fig. S2A) and sequenced individually. In addition, to maximize the diversity investigated, 21 *Haematococcus* strains, including the six abovementioned strains, were inoculated with *P. sedebokerense* PS1 (Fig. S2B), for the DNA of these 21 cultures to be pooled in equal amounts for DNA sequencing. For each inoculated culture, a control (algal culture without fungal inoculum) was performed in parallel and incubated in the same conditions. The time point chosen for harvest (4 dpi) corresponded to a time, where all cultures were visibly infected under the microscope yet before extensive death of algal cells occurred: in this way, we aimed to maximize the differences between bacteria directly involved in the infection of *Haematococcus* by *P. sedebokerense* at the expense of saprophytic bacteria that would thrive on dead algal cells.

#### Inoculation of algal cultures by *P. sedebokerense*

20 ml of 2-week-old *Haematococcus* cultures were then diluted at a ratio 1:10 into fresh 3N-BBM+V in a final volume of 150 ml and incubated as above for 3 weeks.

2.5 ml of the *P. sedebokerense* (strain PS1) inoculum described above was added to 35 ml of the 3-week-old *Haematococcus* culture in a 50 ml Falcon tube. For the pool experiment, 750 μl of the PS1 inoculum was added to 10 ml of a 3-week-old *Haematococcus* culture in a 15 ml Falcon tube. All samples were gently shaken by hand right after inoculation and incubated at 25°C, light intensity of 80 μmol photons m^−2^ s^−1^, 12 h:12 h light–dark period. An aliquot of all samples inoculated with *P. sedebokerense* and the corresponding controls was taken and fixed in 4% paraformaldehyde (PFA) for later observations under the microscope.

#### Sample harvest and DNA extraction

To avoid losing material and remove all the supernatant, all cultures (inoculated and controls) were centrifuged twice to form a dense pellet. First, the 50 ml and 10 ml Falcon tubes were centrifuged 10 min at 10 000 *× g*. Supernatant was then removed leaving ~2 ml in each Falcon. The pellet was resuspended in the remaining supernatant, transferred into a 2 ml Eppendorf and centrifuged again 5 min at 16 863 *× g* and all supernatant discarded. Pellets were flash-frozen in liquid nitrogen and stored at −80°C until being freeze dried. DNA extraction was performed using the Qiagen DNA Plant Easy Kit (Qiagen, Valencia, CA, USA). Briefly, 400 μl of lysis buffer from the Qiagen DNA Plant Easy Kit was added to the freeze-dried pellet and cells were lysed using a Tissue Lyser II (Qiagen) with a 3 mm tungsten carbide bead, for two cycles of 1 min at 30 Hz and stored on ice in between the cycles. 4 μl of 10 mg ml^−1^ RNase was added. The rest of the extraction was done following the manufacturer’s protocol. DNA concentration of each sample was checked with a Qubit assay (Thermo Fisher). For the pool experiment, 30 ng DNA from each of 21 *Haematococcus* strains was pooled based on the Qubit concentration, resulting in a ‘Control pool’ and an ‘Inoculated pool’. The two *P. sedebokerense* strains PS1 and FD61 were sequenced individually to check their axenicity. For this purpose, 2-week-old fungal cultures in CGM were harvested and subjected to DNA extraction in the same way as for the algal cultures.

#### Metagenome sequencing, assembly, and binning

Library preparation and sequencing was performed by the iGenSeq core facility at Institut du Cerveau, Paris. The Illumina DNA Prep kit was used for library preparation following the manufacturer’s guidelines. The libraries were sequenced in paired end mode (2 × 150 bp) in a Novaseq 6000 (Illumina), resulting in a total of ∼341 GB raw data for the 16 libraries (Table S2). Processing of the metagenomics reads was done by using the MetaWRAP pipeline (Uritskiy et al. 2018) along with other bioinformatic tools. Raw reads were preprocessed with the metaWRAP:: Read_qc module, with default settings and including the bm-tagger step to remove any potential human DNA contamination reads. The quality of the sequenced data was checked with FastQC (Andrews et al. 2010) and duplicate reads were removed using fastp (Chen et al. 2018a). The deduplicated paired-end reads were *de novo* assembled using the MEGAHIT assembler implemented in the metaWRAP:: Assembly module (Li et al. 2015). Binning of the assembled genomes was performed with the metaWRAP:: Binning module by running CONCOCT (Alneberg et al. 2014), MaxBin2 (Wu et al. 2016, p. 201), and metaBAT2 (Kang et al. 2019) separately. Using the metaWRAP:: Bin_refinement modules, the bins from the three binning algorithms were refined to produce the best single bin set based on user-provided minimum bin completion and maximum bin contamination parameters. Here, the minimum completion was set to 50% and the maximum allowable contamination rate was set to 10% (i.e. command line -c 50 -x 10). Binning statistics are summarized in Table S3. The refined bins were reassembled my mapping them back on the assembly to further improve the bin set by using the metaWRAP:: reassemble_bins module. The reassembled bins were considered as the final bins, also named Metagenome-Assembled Genomes (MAGs). The metaWRAP:: quant_bins module was used to quantify the abundance of each MAG within a library (i.e. within a sample). Inputs to this module are the nonreassembled bins, the clean reads, and reads from the assembly that are nonbinned. Quant_bins uses Salmon (Patro et al. 2017), a tool used for transcript quantification. The bin abundances output from quant_bins are expressed as ‘genome copies per million reads’ and already normalized by the library size. Metagenome assembly of *P. sedebokerense* strains PS1 and FD61 was performed using MEGAHIT assembler implemented in the metaWRAP:: Assembly module and compared to the reference genome from NCBI (reference GCA_025602915.1) using the genomic alignment dot plot tool D-GENIES (Cabanettes and Klopp 2018), confirming the absence of any bacterial contaminant in both strains.

#### Taxonomic assignment of bacterial metagenomes and abundance variation during infection

The Genome Taxonomy Database Toolkit (GTDB-Tk; Chaumeil et al. 2019) was used for taxonomic assignments of the MAGs (GTDB-Tk v2.1.1) based on the GTDB database (version 207). Heatmaps of bacterial genera and phylum abundance were computed based on output of the MetaWRAP:: quant_bin module, for the corresponding taxonomic level. Using the vegan package of R, the abundances of bacterial MAGs within each sample were first normalized using the Hellinger transformation (decostand function) and a dissimilarity index matrix between samples was calculated with vegdist, using the Euclidean distance and hierarchical clustering performed with hclust, choosing the ‘average’ agglomeration method. To visualize the results, a heatmap was plotted using the pheatmap function of R (package pheatmap). Further, a linear discriminant analysis (LDA) was implemented using the LEfSe module in a conda environment (Segata et al. 2011), using the following metric:


\begin{eqnarray*}
\frac{{2\,\, \times \mathrm{ MAG}\,\,\textrm{abundance}\,\,\textrm{control}}}{{\left( {\mathrm{ MAG}\,\,\textrm{abundance}\,\,\textrm{control} + \,\,\mathrm{ MAG}\,\,\textrm{abundance}\,\,\textrm{infected}} \right)}},
\end{eqnarray*}

with MAG abundances retrieved from the MetaWRAP:: quant_bin module

We tested data for significant differences between classes, here control and infected samples, setting *Haematococcus* strains of a same microbiota type as replicates (see the section ‘The microbiota of laboratory-cultured *Haematococcus* is structured in three community types’ of the section ‘Results’ for a definition of ‘microbiota types’). Parameters were set as default, except for the LDA cutoff score which was set to 0.

#### Functional analysis

Functional annotation of the MAGs was done using eggNOG-mapper v2 (Cantalapiedra et al. 2021), which relies on Prodigal to perform protein prediction. From these annotations, the KEGG Orthologies (KOs) were used in this study. To calculate the abundance of each KO within a MAG, the function featureCounts was used in R (Liao et al. 2014, p. 201). This program counts reads mapping to genomic features based on annotation files. First, the module MetaWRAP:: Blobology was run one each sample to generate a bam file. The bam files were then used together with the annotation files from eggnog as an input to featureCounts. The number of reads mapping per feature, here KO identifiers, was normalized to 1 kB and 1 million reads of annotated KOs within each library. This normalized read count (NRC) per KO was then used for downstream analysis. Firstly, the NRC was used as an input table for LEfSe to test for statistical significance between control and infected samples. Parameters were set as default, except for the LDA cutoff score, which was set to 0. KEMET (Palù et al. 2022), was used to assess completeness of the KEGG modules, based on KO annotations derived from eggnog annotation files. Within each sample, the NRC of each KO were summed across all MAGs, resulting in a total abundance of each KO per sample. This was used as input for a principal component analysis (PCA).

### Phylogenetic tree and estimation of the overall microbiota diversity

16S rRNA reads from the metagenomic libraries were harnessed, assembled, and first assigned taxonomically using MATAM (Pericard et al. 2018) with default settings. Contigs labelled as unclassified by MATAM were manually checked with BLASTn against GenBank n/r, unveiling either chimera that were discarded or meaningful contigs that were kept for the downstream phylogenetic analysis. The initial taxonomic assignment of the contigs and Sanger-sequenced PCR products was performed by BLASTn against NCBI GenBank n/r; the names were cross-checked and updated as appropriate using the GTDB taxonomy. For instance, the genus *Mesorhizobium* is classified as Rhizobiaceae by GTDB and Phyllobacteriaceae by NCBI. In this study, *Mesorhizobium* was classified as a member of the Rhizobiaceae family. Similarly, the new name of Proteobacteria (NCBI database) is Pseudomonadota and the new name of Bacteroidetes is Bacteroidota. The final taxonomic assignment was confirmed using the phylogenetic tree reconstruction as follows. Contigs containing 16S rRNA genes from the metagenomic analysis were aligned with the 16S Sanger sequences from the cultivated microbiota, using MAFFT with default parameters. For simplicity, only one representative 16S rRNA sequence of each bacterial genus was kept for the final alignment, resulting in 28 sequences. RAxML (version 8.2.11, implemented in Geneious Prime 2021.2.2) was used to compute the tree, with 100 bootstrap replicates. No 16S rRNA gene sequences were available for two genera identified in the metagenomics bins with the GTDB-Tk database: genera JACVCJ01 (unclassified Bacteroidota) and JAFKFH01 (family Ferrovibrionaceae). Thus, for representation purpose, sequences representative for the class *Bacteroidota* and the family Ferrovibrionaceae were chosen. The tree was visualized using Itol v. 6.9 (Letunic and Bork 2024).

To estimate the overall microbiota diversity, the presence/absence matrix of bacterial genera identified in the MAGs of the 6 individually sequenced *Haematococcus* strains was used to plot an accumulation curve and to calculate the corresponding Chao2 index using the specpool function of vegan in R. The latter was compared to the total number of bacterial genera (species richness) identified in the pooled sample. The Shannon index was calculated using the abundance of each MAG obtained from the MetaWRAP:: quant_bin module, using the Entropy function from DescTools package in R.

### Microscopy

Both live and paraformaldehyde-fixed *Haematococcus* cultures (infected or control) were observed using a ZEISS Axio Imager 2. Pictures were recorded using an Axiocam colour camera or 705 mono (Zeiss). Calcofluor white was used to stain the fungal pathogen, at a final concentration of 2.5 μg ml^−1^ following the protocol developed by Strittmatter et al. (2015). Labelling was performed for 10–15 min in the dark. After incubation, the stained culture was mounted on a microscope slide and observed by differential interference contrast and fluorescence microscopy (Zeiss, filter set 49, excitation G365, beam splitter FT395, emission BP445/50).

## Results

### The algal–bacterial microbiota is necessary for the infection of *Haematococcus* by *P. sedebokerense*

**Figure 1.**
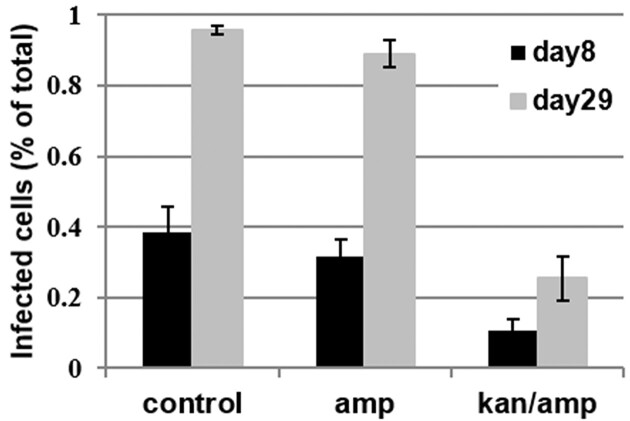
Effect of antibiotic treatments on the infection of *H. pluvialis* strain Haemc1 by *P. sedebokerense* PS1. Ampicillin was used at 150 mg l^−1^ and the combination of ampicillin and kanamycin was used at a concentration of 3.75 mg l^−1^. Cell counts were performed at 8 and 29 days after inoculation. The bars show the mean, and the error bars the standard deviation across four replicates.

A first insight into the importance of the *Haematococcus* microbiota during the infection by *P. sedebokerense* was gained when *H. lacustris* Haemc1 was treated with antibiotics before inoculation with the pathogen (strain PS1). Ampicillin treatment alone did not change the prevalence of infection, but the cocktail of ampicillin and kanamycin reduced the prevalence of infection during the entire time-course, leading to ca. 30% of infected algal cells 29 dpi, whereas nearly all cells were infected in the control *H. lacustris* Haemc1 grown without antibiotic mix (Fig. 1). A dose–response experiment showed that the cocktail of ampicillin and kanamycin at the same concentration reduced the growth rate of a healthy, uninoculated culture of *H. lacustris* Haemc1; however, did not affect the viability of algal cells as judged with Sytox Green labelling according to the protocol of Gerphagnon et al. (2013, data not shown).

**Figure 2.**
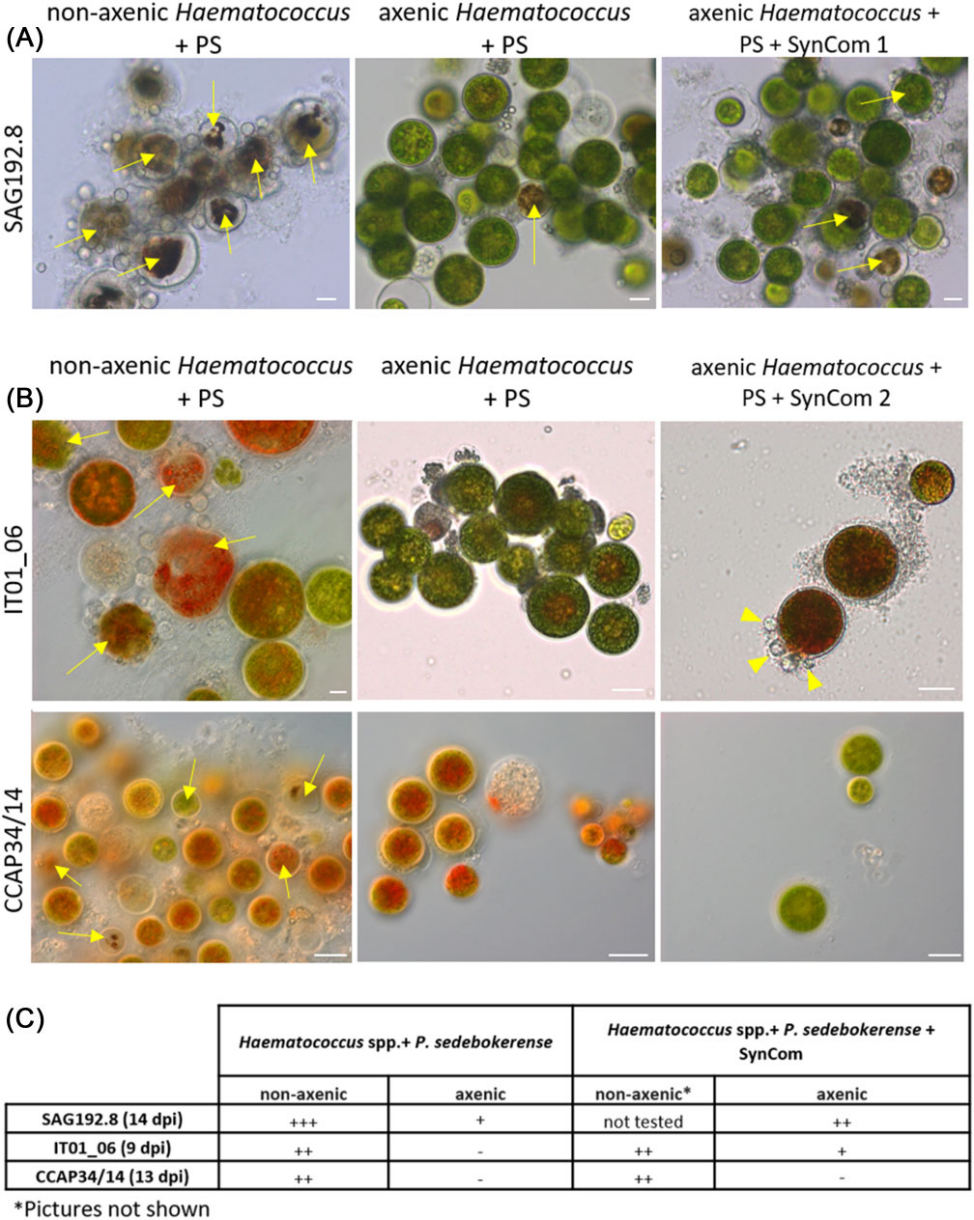
Outcome of the infection by *P. sedebokerense* FD61 of axenic and nonaxenic *Haematococcus* strains, in presence or absence of a bacterial synthetic community (SynCom). (A and B) Microscopy observation. (A) *Haematococcus pluvialis* SAG192.8, 14 days after inoculation. Left: non axenic strain; middle: axenic strain; right: axenic strain supplemented with SynCom 1, a mixture of 10 bacteria from the genera *Mesorhizobium, Aeromicrobium, Microbacterium, Brevundimonas*, and *Variovorax*. (B) *Halictus rubicundus* IT01_06 and *H. pluvialis* CCAP34/14, 9, and 13 days after inoculation, respectively. Arrows: collapsed algal cells; arrowheads: *P. sedebokerense* cysts at the surface of algal cells. Scale bar 10 μm. (C) Qualitative symptom-scoring of the above-described samples, based on a double-blind rating under the microscope: +++: heavily infected algal culture, ++: visible infection symptoms, +: less than 1 < % of infected algal cells, and −: no infection visible. PS: *P. sedebokerense*. The experiment with SynCom 2 was repeated twice independently with identical results.

Next, to more directly investigate the involvement of bacteria in the infection by *P. sedebokerense*, we inoculated the axenic *H. lacustris* strain SAG192.8 with the fungal pathogen (strain FD61). Microscopy observations showed typical infection symptoms in the nonaxenized control with collapsed algal cells turning brown upon advanced stages of infection (Fig. 2A, left panel, arrows). In comparison, almost no infection symptoms were visible in the axenic cultures challenged with *P. sedebokerense* (Fig. 2A and C). Interestingly, a crude attempt to restore the microbiota by adding a synthetic community (SynCom 1) of 10 bacterial strains isolated from the microbiota of *Haematococcus* SAG192.8 almost completely restored the ability of *P. sedebokerense* FD61 to infect this axenic strain (Fig. 2A and C). To further test how general this infection-enabling effect of the microbiota was, two other axenized *Haematococcus* strains, IT01_06 (*H. rubicundus*) and CCAP 34/14 (*H. lacustris*), were used to repeat this experiment, however conducted at another time point and with a simpler SynCom (SynCom 2) made of only one strain of each bacterial genera present in SynCom 1, namely *Mesorhizobium, Aeromicrobium, Microbacterium, Brevundimonas*, and *Variovorax*. As observed previously, no infection symptoms were seen in the axenic cultures challenged by *P. sedebokerense* FD61. When this SynCom 2 was added to axenic IT01_06 and CCAP 34/14, infection of a few algal cells was observed for IT01_06, but not for CCAP34/14 (Fig. 2B and C).

Taken together, these pilot observations show that the bacterial microbiota is required for infection of *Haematococcus* by *P. sedebokerense*, and that its modulation—either with antibiotics or by axenization followed by addition of a synthetic community—affects the outcome of the interaction. The identity of the bacterial player(s) involved, as well as the nature of their interaction with the fungus and/or the alga all remain unknown. Therefore, we set out to undertake a systematic investigation of the diversity of the bacteria associated to *Haematococcus*, and to investigate their changes during infection as well as their metabolic potential and cultivability with the intent to pave the way to identifying specific bacteria (or consortia thereof) suitable for biocontrol of *P. sebokerense*.

### Diversity of the microbiota of laboratory-cultured *Haematococcus*

**Figure 3.**
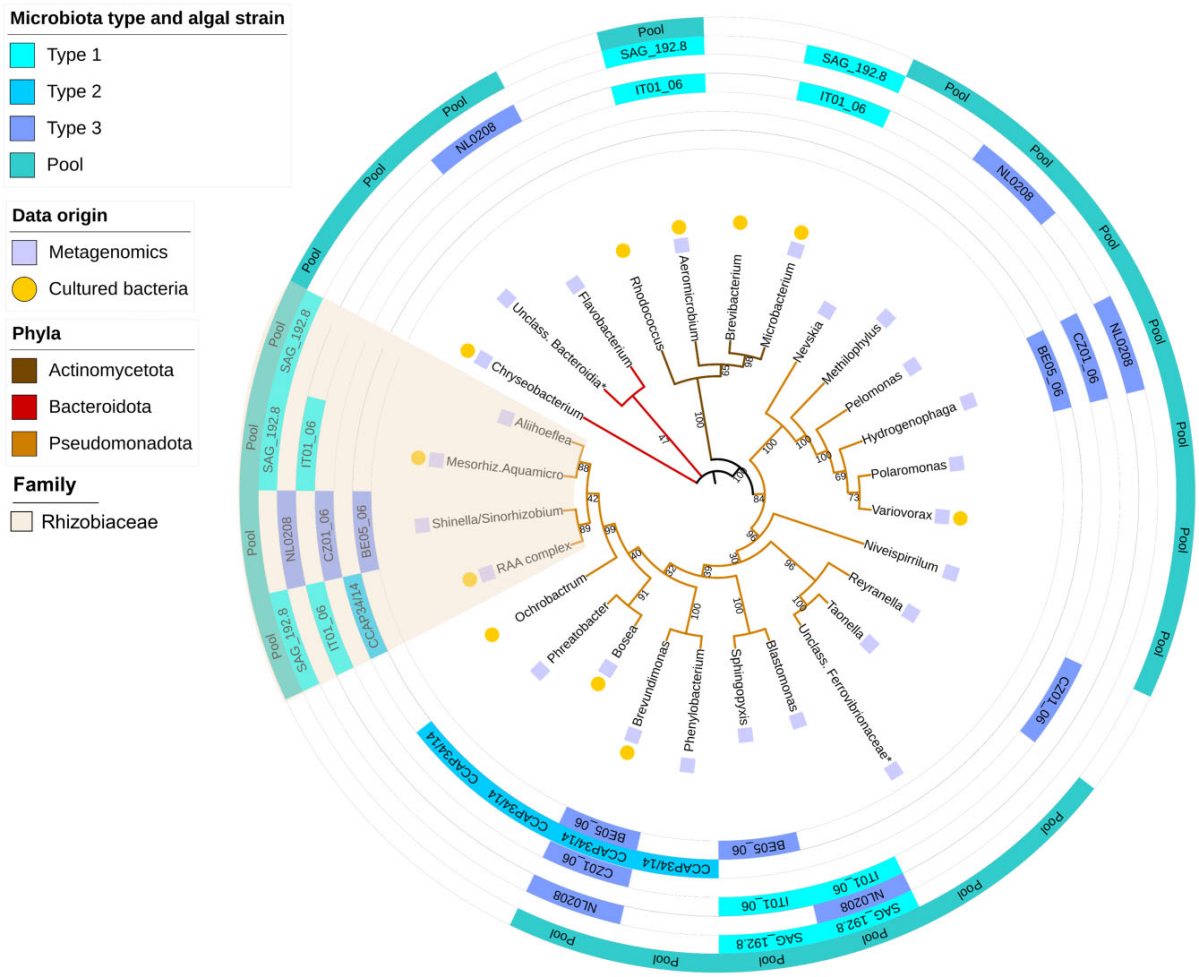
Diversity and phylogenetic relationships of bacterial genera associated to *Haematococcus* based on 16S rRNA sequences. Representative sequences were retrieved from metagenomic reads of the 21 *Haematococcus* strains and from bacteria of the cultivable microbiota from the 44 *Haematococcus* strains. The three 'microbiota types' refer to Fig. 4. Pool: bacteria identified in the pooled DNA of the set of 21 *Haematococcus* strains. RAA complex: *Rhizobium/Agrobacterium/Allorhizobium* complex. Tree branches bearing a * sign represent genera JACVCJ01 (unclassified Bacteroidia) and JAFKFH01 (unclassified Ferrovibrionaceae) as identified with the GTDB-Tk database. In the phylogenetic tree (Fig. 3), some bacterial genera are grouped together because they cannot be distinguished based on 16S (e.g. *Rhizobium, Agrobacterium*, and *Allorhizobium* were grouped into what we called the ‘RAA’ complex). This explains why the tree has 28 branches and not 30.

The microbial community associated to 21 laboratory-cultured *Haematococcus* strains belonging to *H. lacustris, H. rubens*, and *H. rubicundus* (Table S1) was determined by metagenomics (Fig. 3). Of the 21 *Haematococcus* strains, six were investigated individually (Fig. S2). Additionally, a sample containing DNA pooled from the 21 *Haematococcus* strains, named ‘pool’ hereafter, was analyzed in an attempt to capture as much taxonomic and functional diversity as possible. In total, 27 bacterial genera were identified belonging to three phyla: Pseudomonadota, Actinomycetota, and Bacteroidota. Most of the genera (22) belonged to the Pseudomonadota, three genera belong to the Bacteroidota, and two to the Actinomycetota.

In parallel, the cultivable microbiota from 44 *Haematococcus* laboratory-cultured strains including the 21 strains used for metagenomics analysis, were isolated (Table S1). A total of 11 bacterial genera were cultivable and belonged to the same three phyla as identified with metagenomics (Fig. 3). One bacterial genus, *Brucella*, was absent from the metagenomics results, but isolated in the laboratory, most likely because it was isolated from a *Haematococcus* strain not included in the 21 *Haematococcus* strains investigated with metagenomics. The bacterial genera *Brevibacterium* and *Rhodococcus* isolated from *Haematococcus* strains BE05_06 and CCAP34/14, respectively, were not identified using metagenomics. This may be due to DNA extraction for metagenomics and isolation of the cultivable microbiota were not performed at the same time, or that their abundance was below the detection level of our metagenomic investigation. All other cultivable bacteria, however, were detected using metagenomics. The most frequently cultivable bacteria isolated belong to the *Rhizobium/Agrobacterium/Allorhizobium* complex and the genus *Brevundimonas*; they were isolated from 23 and 13 different *Haematococcus* strains, respectively (Tables S4 and S5). All individually studied *Haematococcus* strains included at least one member of the family Rhizobiaceae (Fig. 3, brown disc sector). In total, 30 bacterial genera were identified associated to the set of 44 *Haematococcus* strains, 27 by metagenomics, and 3 more from the cultivable microbiota.

To visualize the overall bacterial diversity associated to the 21 *Haematococcus* strains analyzed by metagenomics, an accumulation curve based on the cumulative number of nonredundant genera identified within the MAGs was calculated (Fig. S3). Additionally, the Chao2 species richness index was calculated to estimate the number of missing genera based on the number of identified genera, resulting in an estimated absolute richness of 25.9 ± 8.6 genera. In comparison, the diversity that we effectively retrieved, including with the pool, is 27 bacterial genera; this suggests that our sampling strategy has achieved a fairly comprehensive, if not exhaustive coverage of the bacterial diversity associated to our *Haematocccus* laboratory cultures.

### The microbiota of laboratory-cultured *Haematococcus* is structured in three community types

**Figure 4.**
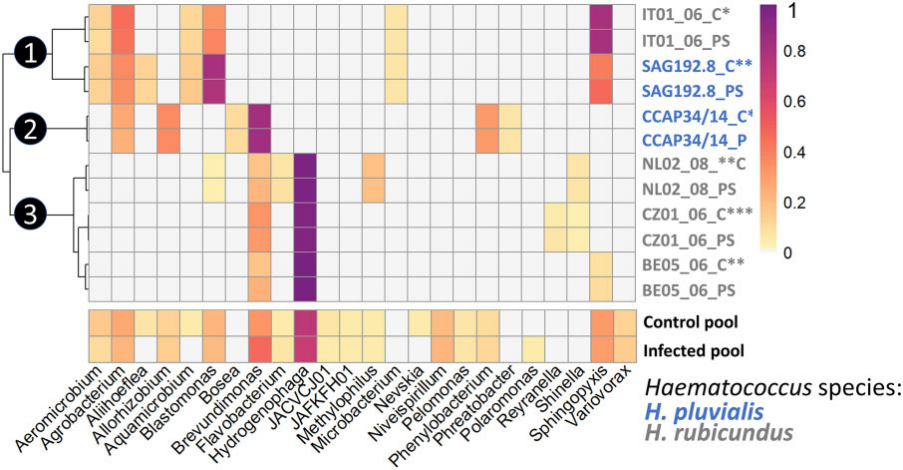
Heatmap of the bacterial genera identified by metagenomics across the 21 *Haematococcus* strains from two species (see legend in the figure) when inoculated with the pathogen *P. sedebokerense* (PS) or in the control condition (C). The heatmap is based on bin abundance per sample normalized by the size of each library, i.e. the total number of reads in each sample. The colour scale corresponds to the abundance matrix normalized using Hellinger transformation. Dissimilarity matrix used for hierarchical cluster analysis was calculated from Euclidean distances. Pooled samples were not included in the clustering but the colour scale was the same as for the individually sequenced samples. 1: Type 1 microbiota, 2: Type 2 microbiota, and 3: Type 3 microbiota. *: low susceptibility, **: intermediate susceptibility, and ***: high susceptibility based on Allewaert et al. (2018) (Fig. S4).

The relative abundance of each bacterial taxon within the six individually sequenced *Haematococcus* strains and the pool were analyzed (Fig. 4). Hierarchical clustering of the corresponding abundance matrix for each bacterial genus splits the algal strains into three types of bacterial communities, each characterized by almost the same genera, and barely overlapping between types. Type 1 microbiota were dominated by the genera *Sphingopyxis, Blastomonas*, and *Agrobacterium*; Type 2 by *Brevundimonas* and *Allorhizobium*; and type 3 by *Hydrogenophaga* and *Brevundimonas*. No bacterial genera were found throughout all the *Haematococcus* strains. The bacterial genus most frequently identified was *Brevundimonas*, as it was present in four out of the six individually analyzed *Haematococcus* strains. At the family level, the most abundant bacteria belong to Burkholderiaceae, which dominates microbiota Type 3, Caulobacteraceae which dominates microbiota Type 2 and Sphingomonadaceae which dominates microbiota Type 1. Rhizobiaceae represent an apparent core family of *Haematococcus*, as representatives of this family were present in all individual *Haematococcus* strains (Fig 3. and Fig. S4). One bacterium belonging to *Haematococcus* strain BE05_06 was identified as being a member of the *Shinella*/*Sinorhizobium* complex (Rhizobiaceae) during the phylogenetic tree reconstruction based on 16S from the metagenomics reads, but the corresponding MAG was not assembled during the metagenomic analysis. This explains why the member *Shinella*/*Sinorhizobium* appears on the phylogenetic tree (Fig. 3) but not on the heatmap (Fig. 4), which was based on the MAGs.

The three microbiota types were found again when the clustering was performed at higher taxonomic levels such as family, order, and class. At the phylum level, another structure was visible (Fig. S5) with three different clusters, all dominated by the phylum Pseudomonadota. Bacteroidota bacteria are only present on strain NL02_08 and Actinomycetota on *Haematococcus* strains IT01_06 and SAG192.8 (microbiota type 1). Every other higher-level taxon was missing from at least one type of microbiota described above. There was no correlation between the *Haematococcus* species and the type of microbiota. Similarly, the observed clustering did not correlate with the contrasting levels of susceptibility of the algal strains to *P. sedebokerense* infection, as determined by Allewaert et al. (2018) (Fig. S6).

**Figure 5.**
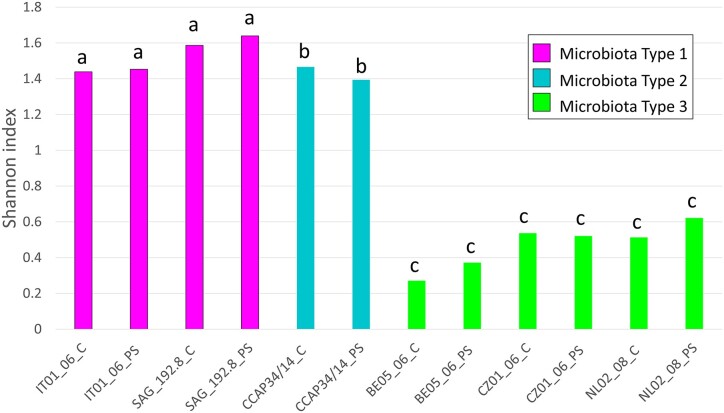
Shannon index calculated at the bacterial genus level for the six individually analyzed *Haematococcus* spp. strains for control (C) and infected samples (PS). The Shannon index was calculated using the abundance of each MAG obtained from the MetaWRAP:: quant_bin module.

Calculation of the Shannon α-diversity index at the genus level (Fig. 5) for each of the *Haematococcus* strains showed a difference in bacterial diversity across the samples. Strains IT01_06, SAG_192.8 (Type 1), and CCAP34/14 (Type 2) have a significantly more diverse microbiota than strains BE05_06, CZ01_06, and NL02_08 from microbiota Type 3 (Shannon index ∼1.5 and ∼0.44, respectively).

### The composition of the *Haematococcus* microbiota is remarkably stable during infection by *P. sedebokerense*

**Figure 6.**
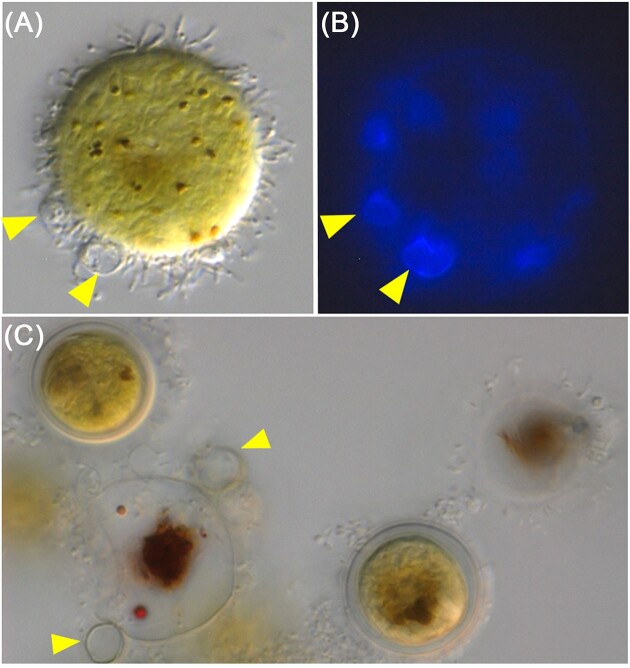
Microscopy images of the *Haematococcus* cultures inoculated with *P. sedebokerense* for the metagenomics experiment showing visible signs of infection. *Paraphysoderma sedebokerense* cells are visible at the algal surface (arrowheads). *Haematococcus* strain CCAP34/14 (A) and (B) calcofluor white staining highlights the presence of the fungal pathogen, (C) *Haematococcus* strain CZ01_06. Cultures were fixed in PFA. Scale bar 10 μm.

To identify bacteria potentially interfering with the *Haematococcus*–*P. sedebokerense* interaction, we investigated the changes of the *Haematococcus* bacterial community upon infection by *P. sedebokerense*. To confirm that infection by *P. sedebokerense* had occurred in the samples used for metagenomic analysis, aliquots of inoculated algae were observed under the microscope. Images show typical disease symptoms, with *P. sedebokerense* cysts developing rhizoids on algal cells for all strains used for metagenomics (Fig. 6 and Fig. S7). As the *P. sedebokerense* inoculum was confirmed to be axenic by metagenomic sequencing, all changes in bacterial composition between the control and infected algal samples can be solely attributed to the algal microbiota. Hierarchical clustering of the bacterial relative abundance grouped all infected samples with their respective control because all bacterial taxa were conserved upon infection and their relative abundance was generally stable (Fig. 4). To investigate quantitative changes in taxa abundance between infected and control samples, we used the LDA LEfSe (Segata et al. 2011), which identifies features (here bacterial taxa) that are differentially abundant between classes, here: ‘infected’ or ‘control’. No significant changes for any bacterial genera (and any higher taxonomic level) were observed between the conditions ‘infected’ or ‘control’ for any of the six algal strains. Running this test independently for each microbiota type did not highlight any significant changes either.

### The functional profile of the *Haematococcus* microbiota is remarkably stable during infection by *P. sedebokerense*

The functional profile of the microbiota of the six individually sequenced *Haematococcus* strains and the pool were analyzed. A total of 4091 unique bacterial KOs, attributed to 329 KEGG pathways (maps) were identified in the MAGs of the six individual *Haematococcus* strains and their relative abundance calculated; likewise, a total of 4362 unique KOs, attributed to 348 KEGG pathways (maps) were found in the MAGs of the pool sample.

The differential abundance analysis with LEfSe across the six individually sequenced strains did not reveal any biological functions, here represented by bacterial KOs, as significantly enriched in any of the control or infected samples (data not shown). Grouping the samples into microbiota type did not highlight differentially abundant functional features neither. Finally, no discriminant functional feature was found between the infected pool with the control pool. Overall, this points to a remarkable stability of the *Haematococcus* microbiota during infection, not only from the taxonomic but also from the functional point of view.

### The functional profiles of the *Haematococcus* microbiota distinguish the three taxonomic microbiota types

**Figure 7.**
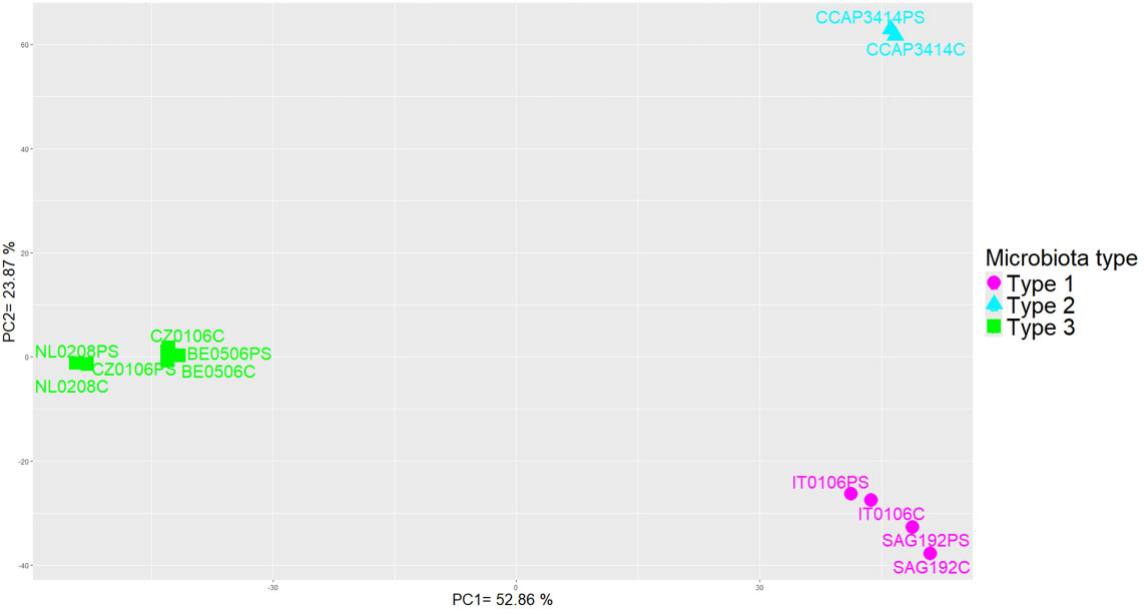
Results of the PCA based on the matrix with the number of reads mapping on annotated features (KOs), normalized to 1 kB and per million read of annotated KOs within each library (sample) and then summed per KO for all MAGs within a library (sample). Projection on the first and second components.

Ordination (PCA) of the total abundance of each bacterial KO per *Haematococcus* sample revealed three functionally distinct groups mapping to the three microbiota types defined in the taxonomic analysis (Fig. 7). The PCA also confirmed the absence of functional separation between any of the infected samples and its corresponding noninfected control. A PCA performed on KO grouped into maps led to the same pattern (Fig. S8).

**Figure 8.**
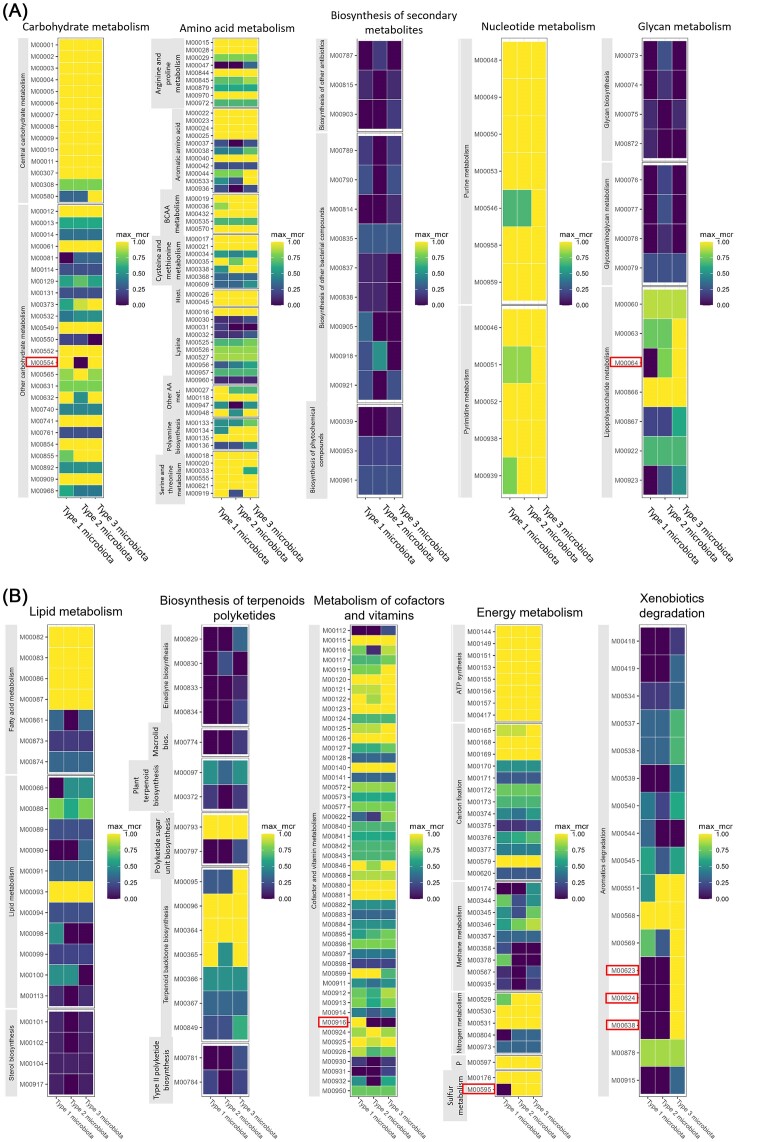
Maximum module completion ratio for each KEGG module within a microbiota type based on KEMET output (Palù et al. 2022). Modules encircled: ‘Nucleotide sugar biosynthesis, galactose ⇒ UDP-galactose’ (M00554), ‘Pyridoxal-P biosynthesis’ (M00916), ‘Thiosulfate oxidation by SOX complex’ (M00595), ‘ADP-l-glycero-d-manno-heptose biosynthesis’ (M00064), and ‘Phthalate, Terephthalate and Salicylate degradation’ (M00623, M00624, and M00638). Abbreviations used: AA: amino acids, BCAA: branched chain amino acids, Histi.: histidine, and P: photosynthesis.

To identify which biological functions were shared across the microbiota types and which ones could explain their separation, the completeness of KEGG modules was assessed. For the six individual *Haematococcus* samples, the completeness of a total of 312 KEGG modules was evaluated based on KOs. KEGG modules were grouped into higher level categories such as: ‘Amino acid metabolism’, ‘Carbohydrate metabolism’, ‘Metabolism of cofactors and vitamins’, and so on, based on KEGG classification. To compare between the three microbiota types, the maximum completion of each module for all taxa identified within a microbiota type was calculated, establishing the cumulative metabolic capacity for each microbiota type. Overall, the communities were very similar to each other (Fig. 8). The pairwise difference in module completeness was calculated between the three microbiota types; modules with a completeness delta of >0.5 were retained as they might explain the most differences between the microbiota type functions. The 16 modules meeting this condition are listed in Table S6. Among them, 7 modules were totally absent from one or two of the three microbiota types (delta equals 1), which implies that these are not vital for algal survival. For instance, the module for ‘Nucleotide sugar biosynthesis, galactose ⇒ UDP-galactose’ (M00554) was absent in the microbiota Type 2 and complete in the two others. The module for ‘Pyridoxal-P biosynthesis’ (M00916) was absent in microbiota Types 2 and 3, and complete in microbiota Type 1. Modules for ‘Thiosulfate oxidation by SOX complex’ (M00595) and ‘ADP-l-glycero-d-manno-heptose biosynthesis’ (M00064) were absent in microbiota Type 1 but almost complete in Types 2 and 3. Finally, modules related to ‘Phthalate, Terephthalate, and Salicylate degradation’ (M00623, M00624, and M00638) were absent from Types 1 and 2 and complete for Type 3.

**Figure 9.**
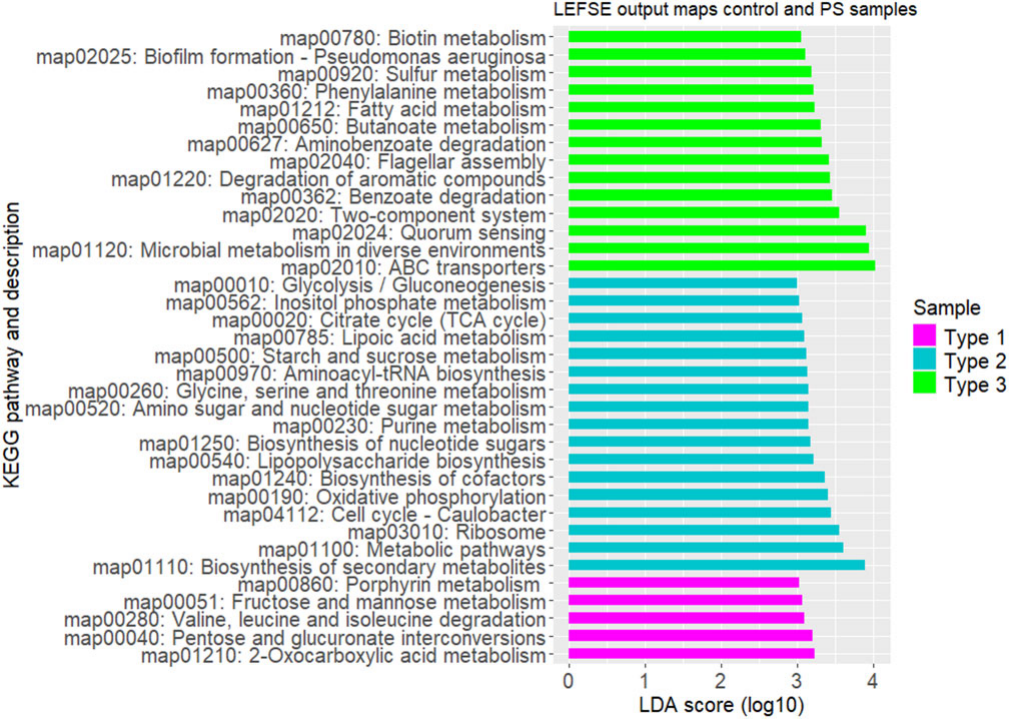
Differences of functional potential between the three microbiota types. The barplot shows the differentially abundant KEGG pathways between microbiota types, based on LDA (*P* < .05 for Kruskal–Wallis test). Only KEGG pathways with LDA score >3 are shown here. The analysis is based on the number of reads mapping on annotated KOs, normalized to 1 kB and 1 million read of annotated KOs within each library, summed per KEGG pathway and then per sample. Here, classes were ‘Microbiota Type’. Control and infected samples were considered as replicates.

To understand the specificities of the three microbiota types at the scale of metabolic pathways (*i.e*. maps), the NRC for each KO were summed across KEGG pathways and then per sample. LEfSe analysis on this table (class set to Microbiota Type) revealed 36 maps that significantly differentiate the three microbiota types (Fig. 9).

## Discussion

### Suppression or modulation of the *Haematococcus* microbiota affects the infection of *Haematococcus* spp. by *P. sedebokerense*

Here, we show that bacteria are necessary for the infection of *Haematococcus* by *P. sedebokerense*, and that modulating the algal microbiota with antibiotics, or the addition of synthetic communities can affect the outcome of the infection. To the best of our knowledge, a requirement for host-associated bacteria is unknown for any algal pathogen. In the fields of medicine and plants however, there is evidence that bacteria promote the virulence of some fungal or oomycete pathogens. For example, biofilms can promote *Candida albicans* infection in humans (Kojic and Darouiche 2004); bacterial exudates promote infection of soybean by the oomycete *Phytophthora nicotianae*; and root-associated bacteria promote the infection of tomato by *Phytophthora parasitica* (Kong and Hong 2016, Larousse et al. 2017). Though rare in the literature, these examples provide useful hypotheses for potential mechanisms, such as the production of signalling molecules, or beneficial trophic interactions.

Beneficial trophic interactions between chytrid and bacteria are recognized in aquatic ecosystems, usually in connection to increased degradation of particulate organic matter and changes in algae-derived dissolved organic matter (DOM), leading to higher bacterial abundance in infected cultures (Senga et al. 2018, Roberts et al. 2020). Another scenario described by Senga et al. 2018 was in the tripartite system of diatom, bacteria, and a chytrid pathogen. They showed that chytrid infection stimulated the release of algal DOM, which led to an increase in bacterial abundance. Hence, a hypothesis to explain why the presence of bacteria is necessary for the fungal infection of *Haematococcus* would be that *P. sedebokerense* benefits from bacterial-derived carbon sources to proliferate. Indeed, this fungal pathogen is routinely cultivated as a saprotroph in CGM media containing yeast extract, peptone, and d-glucose and is known to utilize various carbon sources with a preference for mannose, glucose, and their oligosaccharides (Lin et al. 2021). A nonexclusive hypothesis would be that bacteria associated to *Haematococcus* supply the fungus with secondary metabolites or virulence factors, that may even have some signalling function; accordingly, their identification would offer an insight into possible avenues for interfering with such infection-promoting interactions, and therefore into managing the pathogen in industrial algal cultures.

Conversely, there is a growing number of examples where host-associated bacteria have the opposite effect and inhibit fungal or bacterial pathogens. For example, a secondary metabolite (phenazine-1-carboxamide) produced by *Pseudomonas piscium*, a member of the wheat microbiota, has been shown to modulate histone acetylation in the fungal pathogen *Fusarium graminearum*, thus inhibiting its growth and infectivity (Chen et al. 2018b). Several other mechanisms are reported, including: mycotoxin degradation, induction of the plant defences, or cell wall degradation (Chen et al. 2018b and references therein). Likewise, Lee et al. (2021) were able to induce dysbiosis of the bacterial community in diseased tomato rhizosphere, resulting in increased disease incidence, leading them to conclude that bacterial members of the microbiota might act together or alone as a protection upon infection, and disruption of these interactions could suppress resistance. Comparable examples concerning zoosporic fungal pathogens are few, yet they offer a proof of concept that similar interactions may occur in our system and could be successfully exploited for biocontrol. The most widely studied model is *Batrachochytrium dendrobatidis*, an often fatal chytrid pathogen for many amphibian species (Myers et al. 2012, Park et al. 2014). Bacterial compounds such as violacein, indole 3-carboxaldehyde, or 2,4-diacetylphloroglucinol were shown to inhibit the pathogen’s growth (Brucker et al. 2008, Myers et al. 2012). Antimicrobial peptides from the frog *Rana muscosa*, combined with 2,4-diacetylphloroglucinol, a metabolite from the bacteria *Pseudomonas fluorescens*, act in synergy to inhibit the growth of the *B. dendrobatidis* (Myers et al. 2012). Bacteria isolated from the skin of the boreal toad showed inhibitory activity against the fungus, which correlated with the ability of this species to coexist with the chytrid (Park et al. 2014). This growing body of knowledge on the amphibian skin microbiome has led to encouraging observations of bacteria-mediated enhanced resistance of amphibians to infection: this includes, for example, the inoculation in the environment of bacteria-producing beneficial compounds that get successfully incorporated in the skin microbiota (Muletz et al. 2012), or the treatment of amphibian skin with molecules derived from *B. dendrobatidis* that mimick infection and modulate the skin microbial community towards enhanced resistance (Siomko et al. 2023).

### Members of the *Haematococcus* microbiota are commonly known microalgal associates

The three main bacterial phyla identified in this study, i.e. Pseudomonadota (former Proteobacteria), Bacteroidetes, and Actinomycetota were found in other freshwater green microalgae, including *B. braunii, Scenedesmus quadricauda, Micrasterias crux-melitensis, Nannochloropsis* sp., *Chlorella vulgaris*, and *Chlorella saccharophila* (Table S7) to cite but a few (Ramanan et al. 2015, Krohn-Molt et al. 2017, Blifernez-Klassen et al. 2021). All the bacterial families identified in our work, except *Phreatobacteraceae*, were identified in the abovementioned studies. Among the 30 bacterial genera identified from the collection of the 44 *Haematococcus* strains studied here, 17 (∼50%) of them were also identified associated with in the abovementioned microalgae other than *Haematococcus* (83 bacterial genera in total) (Table S7). 12 bacterial genera (∼10%) were previously identified in other studies investigating the *Haematococcus* microbiota (112 bacterial genera in total) (Table S7). Interestingly, the most frequently identified genus in the other *Haematococcus* studies was *Pseudomonas*, but we did not detect it in our cultures (Table S7). To sum up, among the 30 bacterial genera identified here, 20 were already identified associated to freshwater green microalgae, including *Haematococcus*. The 10 remaining bacterial genera were all reported in aquatic environments. Members of *Aeromicrobium, Brevibacterium, Aquamicrobium*, and *Aliihoeflea* were isolated from seawater or tidal sediments, member of *Niveispirillum* from cyanobacterial aggregates in a eutrophic lake and members from *Pelomonas, Phreatobacter*, and *Taonella* were isolated from water treatment facilities. Additionally, the two unclassified bacteria Bacteroidota (genus JACVCJ01) and Ferrovibrionaceae (JAFKFH01) identified in this study were isolated from an algal culture and a bioreactor inoculated with activated sludge according to NCBI. Overall, the members of the *Haematococcus* microbiota identified here seem to be commonly known algal associates under laboratory and natural conditions. Remarkably, all the green microalgal strains described in Tables S7 and S8, except three strains, were associated to one or more members of the Rhizobiaceae, consistent with previous reports (Pushpakumara et al. 2023). This coassociation of Rhizobiaceae with microalgae is potentially a mutualistic association, as a number of the Rhizobiaceae have been shown to have positive effect on microalgae, such as enhancing algal growth (Kim et al. 2014). The comparison of the abovementioned studies and our data show that the microbiota composition can be highly heterogenous in terms of diversity and composition, even within the same species, such as *H. lacustris* (Table S8). From this, we postulate that culture conditions, origin of isolation have more influence than the microalgal species on the microbiota.

Notably, five of the least abundant bacterial genera detected in the individual algal strains were not found in the pooled sample (which contained the very same individual algal strains), presumably because of limited depth of sequencing. This highlights the complementarity between sequencing deeply a limited number of clonal algal strains and adding a pooled sample, as a compromise between sensitivity and diversity. The resulting estimate of diversity suggests that our sampling strategy enabled to recover most, if not all the taxonomic bacterial diversity associated to the 44 *Haematococcus* strains at hand.

### Structuration and reduction of the microbiota of laboratory-cultured *Haematococcus* provides evidence for its domestication

The microbial composition of our laboratory cultured *Haematococcus* strains clustered in three homogeneous and minimally overlapping groups, highly reminiscent of the ‘microbiome types’ described for plants as discrete or semidiscrete statistical clusters (Toju et al. 2016, 2018). In fact, the concept of ‘microbiome types’ first emerged from medical investigations showing that humans can be grouped into major clusters depending on their gut microbiota, i.e. ‘enterotypes’ (reviewed by Knights et al. 2014). Microbiota types can be conceptualized as alternative stable states which represent, in community theory, locally stable, alternative equilibria of community or ecosystem dynamics.

The fact that similarly structured microbiota are found in *Haematococcus* strains belonging to the same or different species and from distant geographic origins (e.g. Netherlands NL02_08, Czech Republic CZ01_06, and Belgium BE050_05 in Type 3) suggests that the algal identity and biogeography only plays a limited, if any, role in dictating the structure of the microbiota types that we identified. Interestingly, the *Haematococcus* strains from Type 3 microbiota were all sampled from white plastic surfaces (Table S1) and algal strains from Type 1 microbiota isolated from acid bog pools and puddle rock depressions. Therefore, it is plausible to hypothesize that the environmental conditions where *Haematococcus* were first isolated from has the greatest influence in driving the structuring of its microbiota. This would echo the concept of ‘ecological specificity’, known e.g. for fungal communities adapting to the same soil or plant host physiological environments, and which thus show correlated distribution patterns (Dickie 2007).

Importantly, the number of bacterial genera identified in our set of 21 laboratory cultured *Haematococcus* strains was generally lower than has been described for *Haematococcus* or other green algae harvested in the environment (Table S8). Even though the original diversity of the microbiota of our *Haematococcus* strains at the time of isolation was not investigated, our data are therefore consistent with previous observations, which directly showed that microalgal microbiota undergo a reduction in diversity during prolonged cultivation in the laboratory (Krohn-Molt et al. 2017, Behringer et al. 2018, Kublanovskaya et al. 2020), yet retain a specific signature of the original habitat upon domestication (Ajani et al. 2018). Therefore, we hypothesize that our culture conditions (long-term maintenance on solid media, numerous subcultures, controlled light, temperature, and so on) have created a selection pressure that led to a nonrandom loss of the bacterial diversity originally present in environmental samples. This implies that similar microbiota observed in our *Haematococcus* laboratory cultures (e.g. NL02_08, CZ01_06, and BE05_06) might have been quite dissimilar originally, yet have converged towards small communities resembling each other at higher bacterial taxonomic levels. Comparable reduced microbiota types start being identified in plants as a result of interactions between the genotypes and microbiota assembly from soil bacteria: typically the microbes associated to different cultivars tend to be structured around keystone species that play an important functional role with the plant physiology (Toju et al. 2018). In contrast to plants however, the microbiota of algal cultures like ours is inherited vertically and therefore undergoes a domestication process as the direct result of the selection pressures exerted in the laboratory. Emerging evidence on different models shows that these algal microbiota tend to be stable over time (Behringer et al. 2018), that their diversity is reduced in photobioreactors over wild environment (Kublanovskaya et al. 2020), and even more reduced in smaller vessels (Fulbright et al. 2018).

Altogether, the implications of the above is that any of the three reduced microbiota types that we observed would be close to a minimal community of putatively keystone bacteria interlinked together and/or with their *Haematococcus* host by physiologically meaningful biochemical or functional interactions. Therefore, as in other macroorganisms, each reduced microbiota type is potentially a powerful tool to dissect key biochemical and functional interactions within the microbiota and with its host (Banerjee et al. 2018).

### The microbiota of lab-cultured *Haematococcus* is remarkably resilient during infection by *P. sedebokerense*

Despite visible signs of infection by *P. sedebokerense*, the relative abundance of bacterial taxa within the *Haematococcus* microbiota remained stable throughout the infection cycle. This observation was initially surprising given the numerous studies describing shifts in the microbiota of green algae upon biotic and abiotic stresses, including infection by pathogens, over a comparable time scale (e.g. Chekanov et al. 2021). The fact that plants recruit beneficial microbes to face various threats has been widely demonstrated in both land and water ecosystems (Bulgarelli et al. 2015, Toju et al. 2018, Blifernez-Klassen et al. 2021, Gao et al. 2021). For example, disease suppressive soils are being thoroughly investigated to identify bacteria and functions related to the disease suppression mechanism (Ding et al. 2023, Russ et al. 2024). Most of the time, pathogen infection of a plant results in a shift of its associated bacterial community: to cite a few examples, chili pepper microbiome was affected by *Fusarium* sp. (Gao et al. 2021), and the diversity of bacterial endophytes was higher in resistant peach cultivars after inoculation of *Agrobacterium tumefaciens* responsible for the crown gall disease (Li et al. 2019). The same observation was made by Hoeger et al. (2021) in aquatic environment: infection by the aphelid *Amoeboaphelidium protococcarus* of four freshwater microalgae resulted in an increase in bacterial diversity. The functional profile of these bacteria shifted to functions linked to detoxification, degradation, and cellulolysis. Additionally, a proteomic analysis of *S. vacuolatus* infected by the aphelid pathogen revealed an upregulation of proteins related to degradation of small peptides, uptake of a variety of small molecules and several proteins related to bacterial pathogenic interactions with plant hosts (Hoeger et al. 2022). This example indicates that associated bacteria involved in commensal or mutualistic interactions might switch to opportunistic lifestyles and facilitate pathogenic or saprotrophic traits in infected cultures. All this hints at an adapted metabolic pattern to use nutrients probably from algal origin following the pathogenic attack Hoeger et al. (2021, 2022). However, in our case a remarkable stability is observed, both taxonomic and functional, and literature reporting a stability in the microbiota upon pathogen infection is scarce. Baltar et al. (2016) report that microbiota stability when monitoring bacterial marine communities under the predation pressure of grazing protists. This is probably the result of different drivers though, possibly the lack of selectivity of grazers towards particular bacterial taxa. Here, the reduced, possibly even minimal bacterial communities observed in our experiments are likely more resilient to perturbation because of the intensity of the selection pressures that they have already withstood whilst in laboratory culture; therefore, the remaining communities would be less responsive than complex, environmental communities to another stress, such as pathogen infection.

### Functional potential of the *Haematococcus* microbiota types, and their culturablity: a biocontrol and microbiome engineering perspective

Our aim was to investigate the effect of *P. sedebokerense* infection on the functional profile of the *Haematococcus* microbiota. Given the taxonomic and quantitative stability of the bacterial community, it is unsurprising that the functional changes that we identified in the microbiota of *Haematococcus* were mostly between the types that we defined. None of them appeared obviously relevant to the ongoing infection, especially as we could not detect any correlation between the known level of resistance of each algal strain and its associated microbiota type. As our metagenomic experiment was not designed to functionally differentiate between the microbiota of different algal strains, it is therefore virtually impossible to untangle statistically meaningful bacterial functions typical of each microbiota type from bacterial functions that might be shared stochastically between the limited number of algal strains composing each type. Overall, this means that in our system, identifying bacterial biological functions potentially promoting the resistance of the alga against *P. sedebokerense* cannot easily be done via the differential abundance of functional features during infection, a common method successfully used e.g. by Gao et al. (2021) to determine how *Fusarium* wilt disease in chili peppers is influenced by the bacterial genes involved in detoxification, biofilm formation, and plant–microbiome signalling pathways.

An alternative strategy to interrogate the role of bacteria in the interaction between *P. sedebokerense* and *Haematococcus* would be to pinpoint bacterial metagenomes harbouring functions of relevance for the interaction, and use the axenic fungi, algae, and cultivated bacteria to build synthetic communities. In this context, it is particularly interesting that all but 2 of the 27 bacterial genera identified via metagenomics here are known to be cultivable independently from their host alga. Already, we were able to isolate 10 of them using only two common media (CGM and LB). One hypothesis explaining this important proportion of cultivable bacteria in the microbiota of the algal strains studied here could be that the pressures applied through years of laboratory cultivation, and which led to the observed reduction of the microbiota, also favoured the retention of bacteria that can be cultivated independently from the alga. Whatever the underlying mechanisms, we conclude that though the reduced communities of bacteria associated to laboratory-cultured *Haematococcus* probably only represent a subset of the naturally occurring bacterial taxa, their composition appears biased towards an over-representation of keystone, resilient, and easily maintainable bacterial taxa. Combined with the possibility to cultivate both *Haematococcus* and *P. sedebokerense* axenically, the possibility to isolate and maintain clonally a high proportion of these bacteria opens exciting perspectives to engineer synthetic communities and interrogate systematically the interactions with existing phenotyping methods (Allewaert et al. 2018, Calmes et al. 2020), and identify taxa or biological mechanisms that modulate the outcome of the infection of *Haematococcus* by *P. sedebokerense*. Relevant bacterial functions that could be targeted could for example relate to oxidative stress as it was shown to facilitate the infection of *Haematococcus* by *P. sedebokerense* (Yan et al. 2022). Finally, it is noteworthy that some of the bacterial taxa retrieved here are known to have disease suppressive effects in soil, e.g. *Aeromicrobium* (Shen et al. 2018, Ding et al. 2023). *Caulobacter, Rhizobium*, and *Phenylobacterium* were also found in disease suppressive soil (Ding et al. 2023), whereas *Bosea, Brevundimonas*, and *Flavobacterium* were enriched in amendment-induced disease suppressive soils (Russ et al. 2024) and *Flavobacterium* was identified as a keystone species in disease suppressive soil (Mendes et al. 2023). Whether these genera, and the bacteria specifically associated to *Haematococcus*, have a disease-suppressive effect against *P. sedebokerense* can now be investigated by building synthetic communities. Newly acquired knowledge on the structure of *Haematococcus* microbiota types provides the necessary framework to engineer stable microbiomes that hopefully will be scalable to industrial production. The resemblance of the *Haematococcus* with the microbiota of other green algae gives us hope that any novel finding might also be applicable to additional species of industrial interest.

## Supplementary Material

fiaf011_Supplemental_Files

## Data Availability

Data underpinning the bacterial MAGs are available in GenBank under Bioproject PRJNA1151664. This includes the raw sequence data SRR30976931–SRR30976944, each MAG as a BioSample under the accession numbers SAMN45147727–SAMN45147840. The raw reads and the metagenomic assemblies for the two axenic strains of *P. sedebokerense* (PS1 and FD61) have been submitted to GenBank under Bioprojects: PRJNA1197999 and PRJNA1198714, respectively and programmed to be released as soon as the curator has validated the submission. Most *Haematococcus* spp. strains described in this study were obtained from Allewaert et al. (2015) and are publicly accessible via the CCAP collection in Scotland (https://www.ccap.ac.uk/), the BCCM/DCG diatoms and microalgae collection in Belgium (https://bccm.belspo.be/about-DCG), the NORCCA in Norway (https://norcca.scrol.net/), the NIESS in Japan (https://mcc.nies.go.jp/index_en.html), and SAG in Germany (https://sagdb.uni-goettingen.de/). Please refer to Table S1 for details. The strains BE05_17, BE10_08, CH02_05, CH02_08, HU01_03, HU01_04, and Haem2 can be obtained from the authors upon request. The 16S sequences used for taxonomic assignation of the bacteria associated to the 44 *Haematococcus* strains have been submitted to GenBank PQ773519–PQ773595 on 22 December 2024. The bacterial strains are being deposited in the collection BCCM/LMG Bacteria Collection in Belgium. The two *P. sedebokerense* PS1 and FD61 strains are being deposited in the live fungal culture collection at the MNHN.

## References

[bib1] Abdul Malik SA, Bedoux G, Garcia Maldonado JQ et al. Defence on surface: macroalgae and their surface-associated microbiome. In: Advances in Botanical Research. Amsterdam: Elsevier, 2020, 327–68. 10.1016/bs.abr.2019.11.009.

[bib2] Ajani PA, Kahlke T, Siboni N et al. The microbiome of the cosmopolitan diatom Leptocylindrus reveals significant spatial and temporal variability. Front Microbiol. 2018;9:2758. 10.3389/fmicb.2018.02758. 30498485 PMC6249420

[bib3] Allewaert CC, Hiegle N, Strittmatter M et al. Life history determinants of the susceptibility of the blood alga *Haematococcus* to infection by *Paraphysoderma sedebokerense* (Blastocladiomycota). Algal Res. 2018;31:282–90. 10.1016/j.algal.2018.02.015.

[bib4] Allewaert CC, Vanormelingen P, Pröschold T et al. Species diversity in European *Haematococcus pluvialis* (Chlorophyceae, Volvocales). Phycologia. 2015;54:583–98. 10.2216/15-55.1.

[bib5] Alneberg J, Bjarnason BS, De Bruijn I et al. Binning metagenomic contigs by coverage and composition. Nat Methods. 2014;11:1144–6. 10.1038/nmeth.3103. 25218180

[bib6] Alors D, Amses KR, James TY et al. *Paraphysoderma sedebokerense* GlnS III is essential for the infection of its host *Haematococcus lacustris*. J Fungi. 2022;8:561. 10.3390/jof8060561. PMC9224648 35736044

[bib8] Alors D, Boussiba S, Zarka A. Drought resistant resting cysts of *Paraphysoderma sedebokerense* preserves the species viability and its virulence. Plants. 2023;12:3230. 10.3390/plants12183230. 37765394 PMC10537327

[bib7] Alors D, Boussiba S, Zarka A. *Paraphysoderma sedebokerense* infection in three economically valuable microalgae: host preference correlates with parasite fitness. J Fungi. 2021;7:100. 10.3390/jof7020100. PMC7912770 33535515

[bib9] Andrews S, Krueger F, Seconds-Pichon A et al. FastQC. A quality control tool for high throughput sequence data. Cambridgeshire: Babraham Bioinformatics, 2010.

[bib10] Baltar F, Palovaara J, Unrein F et al. Marine bacterial community structure resilience to changes in protist predation under phytoplankton bloom conditions. ISME J. 2016;10:568–81. 10.1038/ismej.2015.135. 26262814 PMC4817682

[bib11] Banerjee S, Schlaeppi K, Van Der Heijden MGA. Keystone taxa as drivers of microbiome structure and functioning. Nat Rev Microbiol. 2018;16:567–76. 10.1038/s41579-018-0024-1. 29789680

[bib12] Behringer G, Ochsenkühn MA, Fei C et al. Bacterial communities of diatoms display strong conservation across strains and time. Front Microbiol. 2018;9:659. 10.3389/fmicb.2018.00659. 29681892 PMC5897529

[bib13] Blifernez-Klassen O, Klassen V, Wibberg D et al. Phytoplankton consortia as a blueprint for mutually beneficial eukaryote-bacteria ecosystems based on the biocoenosis of *Botryococcus* consortia. Sci Rep. 2021;11:1726. 10.1038/s41598-021-81082-1. 33462312 PMC7813871

[bib14] Brucker RM, Harris RN, Schwantes CR et al. Amphibian chemical defense: antifungal metabolites of the microsymbiont *Janthinobacterium lividum* on the Salamander *Plethodon cinereus*. J Chem Ecol. 2008;34:1422–9. 10.1007/s10886-008-9555-7. 18949519

[bib15] Bulgarelli D, Garrido-Oter R, Münch PC et al. Structure and function of the bacterial root microbiota in wild and domesticated barley. Cell Host Microbe. 2015;17:392–403. 10.1016/j.chom.2015.01.011. 25732064 PMC4362959

[bib16] Cabanettes F, Klopp C. D-GENIES: dot plot large genomes in an interactive, efficient and simple way. PeerJ. 2018;6:e4958. 10.7717/peerj.4958. 29888139 PMC5991294

[doi89_978_244825] Calmes B, Strittmatter M, Jacquemin B et al. Parallelisable non-invasive biomass, fitness and growth measurement of macroalgae and other protists with nephelometry. Algal Res. 2020;46:101762. 10.1016/j.algal.2019.101762

[bib17] Cantalapiedra CP, Hernández-Plaza A, Letunic I et al. eggNOG-mapper v2: functional annotation, orthology assignments, and domain prediction at the metagenomic scale. Mol Biol Evol. 2021;38:5825–9. 10.1093/molbev/msab293. 34597405 PMC8662613

[bib18] Carney L, Lane TW. Parasites in algae mass culture. Front Microbiol. 2014;8. 10.3389/fmicb.2014.00278. PMC4047527 24936200

[bib19] Carney L, Sorensen K. *Haematococcus pluvialis* culture compositions. 2016. Patent No.: US 9,347,035 B1.

[bib20] Chaumeil P-A, Mussig AJ, Hugenholtz P et al. GTDB-Tk: a toolkit to classify genomes with the Genome Taxonomy Database. Bioinformatics. 2019;36:1925. 10.1093/bioinformatics/btz848. 31730192 PMC7703759

[bib21] Chekanov K, Zaytseva A, Mamedov I et al. The dynamics of the bacterial community of the photobioreactor-cultivated green microalga *Haematococcus lacustris* during stress-induced astaxanthin accumulation. Biology. 2021;10:115. 10.3390/biology10020115. 33557358 PMC7915213

[bib22] Chen S, Zhou Y, Chen Y et al. fastp: an ultra-fast all-in-one FASTQ preprocessor. Bioinformatics. 2018a;34:i884–90. 10.1093/bioinformatics/bty560. 30423086 PMC6129281

[bib23] Chen Y, Wang J, Yang N et al. Wheat microbiome bacteria can reduce virulence of a plant pathogenic fungus by altering histone acetylation. Nat Commun. 2018b;9:3429. 10.1038/s41467-018-05683-7. 30143616 PMC6109063

[bib24] Dickie IA . Host preference, niches and fungal diversity. New Phytol. 2007;174:230–3. 10.1111/j.1469-8137.2007.02055.x. 17388883

[bib25] Ding J, Wang N, Liu P et al. Bacterial wilt suppressive composts: significance of rhizosphere microbiome. Waste Manag. 2023;169:179–85. 10.1016/j.wasman.2023.07.011. 37453305

[bib26] Ding Y, Zhang A, Wen X et al. Application of surfactants for controlling destructive fungus contamination in mass cultivation of *Haematococcus pluvialis*. Bioresour Technol. 2020;317:124025. 10.1016/j.biortech.2020.124025. 32836037

[bib27] Dittami SM, Eveillard D, Tonon T. A metabolic approach to study algal–bacterial interactions in changing environments. Mol Ecol. 2014;23:1656–60. 10.1111/mec.12670. 24447216

[bib28] Fisher CL, Ward CS, Lane PD et al. Bacterial communities protect the alga *Microchloropsis salina* from grazing by the rotifer *Brachionus plicatilis*. Algal Res. 2019;40:101500. 10.1016/j.algal.2019.101500.

[bib29] Fulbright SP, Robbins-Pianka A, Berg-Lyons D et al. Bacterial community changes in an industrial algae production system. Algal Res. 2018;31:147–56. 10.1016/j.algal.2017.09.010. 29785358 PMC5959032

[bib30] Gao M, Xiong C, Gao C et al. Disease-induced changes in plant microbiome assembly and functional adaptation. Microbiome. 2021;9:187. 10.1186/s40168-021-01138-2. 34526096 PMC8444440

[bib31] Gerphagnon M, Latour D, Colombet J et al. A double staining method using SYTOX green and calcofluor white for studying fungal parasites of phytoplankton. Appl Environ Microbiol. 2013;79:3943–51. 10.1128/AEM.00696-13. 23603679 PMC3697587

[bib32] Gutman J, Zarka A, Boussiba S. The host-range of *Paraphysoderma sedebokerensis*, a chytrid that infects *Haematococcus pluvialis*. Eur J Phycol. 2009;44:509–14. 10.1080/09670260903161024.

[bib33] Han D, Li Y, Hu Q. Biology and commercial aspects of *Haematococcus pluvialis*. In: Richmond A, Hu Q (eds), Handbook of Microalgal Culture. Oxford: John Wiley & Sons, Ltd, 2013, 388–405. 10.1002/9781118567166.ch20.

[bib34] Hoeger A-L, Griehl C, Noll M. Infection with intracellular parasite *Amoeboaphelidium protococcarum* induces shifts in associated bacterial communities in microalgae cultures. J Appl Phycol. 2021;33:2863–73. 10.1007/s10811-021-02542-9.

[bib35] Hoeger A-L, Jehmlich N, Kipping L et al. Associated bacterial microbiome responds opportunistic once algal host *Scenedesmus vacuolatus* is attacked by endoparasite *Amoeboaphelidium protococcarum*. Sci Rep. 2022;12:13187. 10.1038/s41598-022-17114-1. 35915148 PMC9343445

[bib36] Hoffman Y, Aflalo C, Zarka A et al. Isolation and characterization of a novel chytrid species (phylum *Blastocladiomycota*), parasitic on the green alga *Haematococcus*. Mycol Res. 2008;112:70–81. 10.1016/j.mycres.2007.09.002. 18222678

[bib37] Hwang S-W, Choi HI, Sim SJ. Acidic cultivation of *Haematococcus pluvialis* for improved astaxanthin production in the presence of a lethal fungus. Bioresour Technol. 2019;278:138–44. 10.1016/j.biortech.2019.01.080. 30685617

[bib38] James TY, Hoffman Y, Zarka A et al. *Paraphysoderma sedebokerense*, gen. et sp. nov., an aplanosporic relative of *Physoderma* (*Blastocladiomycota*). Mycotaxon. 2012;118:177–80. 10.5248/118.177.

[bib39] Kagami M, Van Donk E, De Bruin A et al. *Daphnia* can protect diatoms from fungal parasitism. Limnol Oceanogr. 2004;49:680–5. 10.4319/lo.2004.49.3.0680.

[bib40] Kang DD, Li F, Kirton E et al. MetaBAT 2: an adaptive binning algorithm for robust and efficient genome reconstruction from metagenome assemblies. PeerJ. 2019;7:e7359. 10.7717/peerj.7359. 31388474 PMC6662567

[bib41] Kim B-H, Ramanan R, Cho D-H et al. Role of rhizobium, a plant growth promoting bacterium, in enhancing algal biomass through mutualistic interaction. Biomass Bioenergy. 2014;69:95–105. 10.1016/j.biombioe.2014.07.015.

[bib42] Knights D, Ward TL, McKinlay CE et al. Rethinking “enterotypes”. Cell Host Microbe. 2014;16:433–7. 10.1016/j.chom.2014.09.013. 25299329 PMC5558460

[bib43] Kojic EM, Darouiche RO. *Candida* infections of medical devices. Clin Microbiol Rev. 2004;17:255–67. 15084500 10.1128/CMR.17.2.255-267.2004 PMC387407

[bib44] Kong P, Hong C. Soil bacteria as sources of virulence signal providers promoting plant infection by *Phytophthora* pathogens. Sci Rep. 2016;6:33239. 10.1038/srep33239. 27616267 PMC5018965

[bib45] Krohn I, Menanteau-Ledouble S, Hageskal G et al. Health benefits of microalgae and their microbiomes. Microb Biotechnol. 2022;15:1966–83. 10.1111/1751-7915.14082. 35644921 PMC9249335

[bib46] Krohn-Molt I, Alawi M, Förstner KU et al. Insights into microalga and bacteria interactions of selected phycosphere biofilms using metagenomic, transcriptomic, and proteomic approaches. Front Microbiol. 2017;8:1941. 10.3389/fmicb.2017.01941. 29067007 PMC5641341

[bib47] Kublanovskaya A, Solovchenko A, Fedorenko T et al. Natural communities of carotenogenic chlorophyte *Haematococcus lacustris* and bacteria from the White Sea Coastal Rock ponds. Microb Ecol. 2020;79:785–800. 10.1007/s00248-019-01437-0. 31676992

[bib48] Larousse M, Rancurel C, Syska C et al. Tomato root microbiota and *Phytophthora parasitica*-associated disease. Microbiome. 2017;5:56. 10.1186/s40168-017-0273-7. 28511691 PMC5434524

[bib49] Lee C, Jeon MS, Kim JY et al. Effects of an auxin-producing symbiotic bacterium on cell growth of the microalga *Haematococcus pluvialis*: elevation of cell density and prolongation of exponential stage. Algal Res. 2019;41:101547. 10.1016/j.algal.2019.101547.

[bib50] Lee S-A, Kim M, Esterhuizen M et al. An acceleration of carotenoid production and growth of *Haematococcus lacustris* induced by host–microbiota network interaction. Microbiol Res. 2022;262:127097. 10.1016/j.micres.2022.127097. 35751943

[bib51] Lee S-M, Kong HG, Song GC et al. Disruption of firmicutes and actinobacteria abundance in tomato rhizosphere causes the incidence of bacterial wilt disease. ISME J. 2021;15:330–47. 10.1038/s41396-020-00785-x. 33028974 PMC7852523

[bib52] Letcher PM, Lee PA, Lopez S et al. An ultrastructural study of *Paraphysoderma sedebokerense* (Blastocladiomycota), an epibiotic parasite of microalgae. Fungal Biol. 2016;120:324–37. 10.1016/j.funbio.2015.11.003. 26895861

[bib53] Letunic I, Bork P. Interactive Tree of Life (iTOL) v6: recent updates to the phylogenetic tree display and annotation tool. Nucleic Acids Res. 2024;52:W78. 10.1093/nar/gkae268. 38613393 PMC11223838

[bib54] Li D, Liu C-M, Luo R et al. MEGAHIT: an ultra-fast single-node solution for large and complex metagenomics assembly via succinct de Bruijn graph. Bioinformatics. 2015;31:1674–6. 10.1093/bioinformatics/btv033. 25609793

[bib55] Li Q, Guo R, Li Y et al. Insight into the bacterial endophytic communities of peach cultivars related to crown gall disease resistance. Appl Environ Microbiol. 2019;85. 10.1128/AEM.02931-18. PMC6495757 30824451

[bib56] Lian J, Wijffels RH, Smidt H et al. The effect of the algal microbiome on industrial production of microalgae. Microb Biotechnol. 2018;11:806–18. 10.1111/1751-7915.13296. 29978601 PMC6116740

[bib57] Liao Y, Smyth GK, Shi W. featureCounts: an efficient general purpose program for assigning sequence reads to genomic features. Bioinformatics. 2014;30:923–30. 10.1093/bioinformatics/btt656. 24227677

[bib58] Lin J, Yan H, Zhao L et al. Interaction between the cell walls of microalgal host and fungal carbohydrate-activate enzymes is essential for the pathogenic parasitism process. Environ Microbiol. 2021;23:5114–30. 10.1111/1462-2920.15465. 33723900

[bib59] Liu H, Li J, Carvalhais LC et al. Evidence for the plant recruitment of beneficial microbes to suppress soil-borne pathogens. New Phytol. 2020;229:2873–85. 10.1111/nph.17057. 33131088

[bib60] Mendes LW, Raaijmakers JM, De Hollander M et al. Impact of the fungal pathogen *Fusarium oxysporum* on the taxonomic and functional diversity of the common bean root microbiome. Environ Microbiome. 2023;18:68. 10.1186/s40793-023-00524-7. 37537681 PMC10401788

[bib61] Muletz CR, Myers JM, Domangue RJ et al. Soil bioaugmentation with amphibian cutaneous bacteria protects amphibian hosts from infection by *Batrachochytrium dendrobatidis*. Biol Conserv. 2012;152:119–26. 10.1016/j.biocon.2012.03.022.

[bib62] Myers JM, Ramsey JP, Blackman AL et al. Synergistic inhibition of the lethal fungal pathogen *Batrachochytrium dendrobatidis*: the combined effect of symbiotic bacterial metabolites and antimicrobial peptides of the frog *Rana muscosa*. J Chem Ecol. 2012;38:958–65. 10.1007/s10886-012-0170-2. 22914957

[bib63] Nakada T, Ota S. What is the correct name for the type of *Haematococcus* flot. (Volvocales, Chlorophyceae)?. TAXON. 2016;65:343–8. 10.12705/652.11.

[bib64] Palù M, Basile A, Zampieri G et al. KEMET—a python tool for KEGG Module evaluation and microbial genome annotation expansion. Comput Struct Biotechnol J. 2022;20:1481–6. 10.1016/j.csbj.2022.03.015. 35422973 PMC8976094

[bib65] Park ST, Collingwood AM, St-Hilaire S et al. Inhibition of *Batrachochytrium dendrobatidis* caused by bacteria isolated from the skin of boreal toads, *Anaxyrus (Bufo) boreas boreas*, from Grand Teton National Park, Wyoming, USA. Microbiol Insights. 2014;7:1–8. 10.4137/MBI.S13639. 24826077 PMC4019225

[bib66] Patro R, Duggal G, Love MI et al. Salmon provides fast and bias-aware quantification of transcript expression. Nat Methods. 2017;14:417–9. 10.1038/nmeth.4197. 28263959 PMC5600148

[bib67] Pericard P, Dufresne Y, Couderc L et al. MATAM: reconstruction of phylogenetic marker genes from short sequencing reads in metagenomes. Bioinformatics. 2018;34:585–91. 10.1093/bioinformatics/btx644. 29040406

[bib68] Pushpakumara BLDU, Tandon K, Willis A et al. Unravelling microalgal-bacterial interactions in aquatic ecosystems through 16S rRNA gene-based co-occurrence networks. Sci Rep. 2023;13:2743. 10.1038/s41598-023-27816-9. 36797257 PMC9935533

[bib69] Qin S, Wang K, Gao F et al. Biotechnologies for bulk production of microalgal biomass: from mass cultivation to dried biomass acquisition. Biotechnol Biofuels Bioprod. 2023;16:131. 10.1186/s13068-023-02382-4. 37644516 PMC10466707

[bib70] Ramanan R, Kang Z, Kim B-H et al. Phycosphere bacterial diversity in green algae reveals an apparent similarity across habitats. Algal Res. 2015;8:140–4. 10.1016/j.algal.2015.02.003.

[bib71] Roberts C, Allen R, Bird KE et al. Chytrid fungi shape bacterial communities on model particulate organic matter. Biol Lett. 2020;16:20200368. 10.1098/rsbl.2020.0368. 32991826 PMC7532721

[bib72] Russ L, Andreo Jimenez B, Nijhuis E et al. *Rhizoctonia solani* disease suppression: addition of keratin-rich soil amendment leads to functional shifts in soil microbial communities. FEMS Microbiol Ecol. 2024;100:fiae024. 10.1093/femsec/fiae024. 38499445 PMC10959553

[bib73] Segata N, Izard J, Waldron L et al. Metagenomic biomarker discovery and explanation. Genome Biol. 2011;12:R60. 10.1186/gb-2011-12-6-r60. 21702898 PMC3218848

[bib74] Senga Y, Yabe S, Nakamura T et al. Influence of parasitic chytrids on the quantity and quality of algal dissolved organic matter (AOM). Water Res. 2018;145:346–53. 10.1016/j.watres.2018.08.037. 30170302

[bib75] Seymour JR, Amin SA, Raina J-B et al. Zooming in on the phycosphere: the ecological interface for phytoplankton–bacteria relationships. Nat Microbiol. 2017;2:17065. 10.1038/nmicrobiol.2017.65. 28555622

[bib76] Shen G, Zhang S, Liu X et al. Soil acidification amendments change the rhizosphere bacterial community of tobacco in a bacterial wilt affected field. Appl Microbiol Biotechnol. 2018;102:9781–91. 10.1007/s00253-018-9347-0. 30302520 PMC6208964

[bib77] Siomko SA, Greenspan SE, Barnett KM et al. Selection of an anti-pathogen skin microbiome following prophylaxis treatment in an amphibian model system. Philos Trans R Soc B Biol Sci. 2023;378:20220126. 10.1098/rstb.2022.0126. PMC10258671 37305917

[bib78] Strittmatter M, Guerra T, Silva J et al. A new flagellated dispersion stage in *Paraphysoderma sedebokerense*, a pathogen of *Haematococcus pluvialis*. J Appl Phycol. 2015;28:1553–8. 10.1007/s10811-015-0700-8. 27226700 PMC4851982

[bib79] Strittmatter M, Rad-Menéndez C, Gachon CMM. Cryopreservation of the parasitic and saprophytic life stage of the blastocladialean pathogen *Paraphysoderma sedebokerense* infecting the green algae *Haematococcus pluvialis* and *Scenedesmus dimorphus*. Phycologia. 2020;59:566–70. 10.1080/00318884.2020.1827825.

[bib80] Syed Ab Rahman SF, Singh E, Pieterse CMJ et al. Emerging microbial biocontrol strategies for plant pathogens. Plant Sci. 2018;267:102–11. 10.1016/j.plantsci.2017.11.012. 29362088

[bib81] Toju H, Peay KG, Yamamichi M et al. Core microbiomes for sustainable agroecosystems. Nat Plants. 2018;4:247–57. 10.1038/s41477-018-0139-4. 29725101

[bib82] Toju H, Yamamoto S, Tanabe AS et al. Network modules and hubs in plant-root fungal biomes. J R Soc Interface. 2016;13:20151097. 10.1098/rsif.2015.1097. 26962029 PMC4843674

[bib83] Uritskiy GV, DiRuggiero J, Taylor J. MetaWRAP—a flexible pipeline for genome-resolved metagenomic data analysis. Microbiome. 2018;6:158. 10.1186/s40168-018-0541-1. 30219103 PMC6138922

[bib84] Villaró S, Ciardi M, Morillas-España A et al. Microalgae derived astaxanthin: research and consumer trends and industrial use as food. Foods. 2021;10:2303. 10.3390/foods10102303. 34681351 PMC8534595

[bib86] Wu Y-W, Simmons BA, Singer SW. MaxBin 2.0: an automated binning algorithm to recover genomes from multiple metagenomic datasets. Bioinformatics. 2016;32:605–7. 10.1093/bioinformatics/btv638. 26515820

[bib87] Yan H, Ma H, Li Y et al. Oxidative stress facilitates infection of the unicellular alga *Haematococcus pluvialis* by the fungus *Paraphysoderma sedebokerense*. Biotechnol Biofuels Bioprod. 2022;15:56. 35596207 10.1186/s13068-022-02140-y PMC9123766

[bib88] Yu BS, Lee SY, Sim SJ. Effective contamination control strategies facilitating axenic cultivation of *Haematococcus pluvialis*: risks and challenges. Bioresour Technol. 2022;344:126289. 10.1016/j.biortech.2021.126289. 34748979

