# Supplementary material for: Importance, structure, cultivability, and resilience of the bacterial microbiota during infection of laboratory-grown Haematococcus spp. by the blastocladialean pathogen Paraphysoderma sedebokerense: evidence for a domesticated microbiota and its potential for biocontrol
Source: FEMS Microbiol Ecol. 2025 Jan 20;101(2):fiaf011. doi: 10.1093/femsec/fiaf011 (PMC11797010; doi:10.1093/femsec/fiaf011)
Supplement: fiaf011_Supplemental_Files [file fiaf011_supplemental_files.zip › supplementary_figures_R1.docx]

# Supplementary Tables legends:

**Supplementary Table 1.** List of the of 44 *Haematococcus* strains used in this study. The strains used for the metagenomic analysis are marked in bold (21 strains). Six of these strains, marked with an asterisk were sequenced individually in the metagenomics analysis. The original table was taken from Allewaert et al., (2018).

**Supplementary Table 2.** Reads and assembly statistics. Quality check done by using fastQC, deduplication using fastp and assembly using MEGAHIT. For more details about metagenomic pipeline see Material and Methods.

**Supplementary Table 3.** Binning statistics summarising the outputs of Metawrap. Taxonomy was done using GTDB module based on the GTDB-Tk database.

**Supplementary Table 4.** Overview of the bacterial genera identified with both the metagenomics analysis and the bacteria isolated in the laboratory. n.d: not detected.

**Supplementary Table 5.** List of the bacteria isolated from the collection of 44 *Haematococcus* spp. strains, with their taxonomic identification based on 16S rRNA gene and the corresponding Genbank accession number.

**Supplementary Table 6.** Top 16 KEGG modules that are most different in terms of module completion between the three microbiota types.

**Supplementary Table 7.** Comparative table of the bacterial families and genera previously identified associated with green freshwater microalgae (excluding *Haematococcus* spp.). Data was taken from a non-exhaustive list of five studies. 0 absence, 1 presence of the bacterial genus. Bacterial genera designated as “uncultured bacterium” or “unclassified” in the papers were not including in this table. In bold, bacterial genera that were also identified in the *Haematococcus* spp. strains in this study. ANPR: *Allorhizobium-Neorhizobium-Pararhizobium-Rhizobium*.

**Supplementary Table 8**. Comparative table of bacterial genera from different *Haematococcus* spp. microbiota identified by 16S rRNA sequencing (1-7) or metagenomics (8,9), (1): *H. pluvialis* (unspecified strain) cultivated in open raceway pond bioreactors (Carney and Sorensen, 2015) (2): *H. lacustris* (strains NIES-144 and UTEX 2505) maintained under laboratory conditions (Lee et al., 2019), (3): *H. lacustris* collected on the White Sea Coast (Kublanovskaya et al., 2019) (4) *H. lacustris* maintained under laboratory conditions (Kublanovskaya et al., 2019) (5): *H. lacustris* (strain BM1, IPPAS H-2018) cultivated in a glass column photobioreactor during astaxanthin accumulation (Chekanov et al., 2021) (6): *H. lacustris* (strain NIES-144) maintained under laboratory conditions (Lee et al., 2022) (7) *H. pluvialis* (strain LAMB284) maintained under laboratory conditions, microbiota composition determined at exponential growth stage and stationary stage; in total 73 genera were reported but only the 10 main are named in the paper (Li et al., 2022), (8) this study: set of 6 *Haematococcus* spp. strains maintained under laboratory conditions, (9) this study: set of the 44 *Haematococcus* spp. strains (Supp. Table 1), including bacteria identified by metagenomics and from the cultivable microbiota. Unclassified and uncultured bacteria are not shown in the table. 0 = absence, 1 = presence of the bacterial genus.

# Supplementary figures:


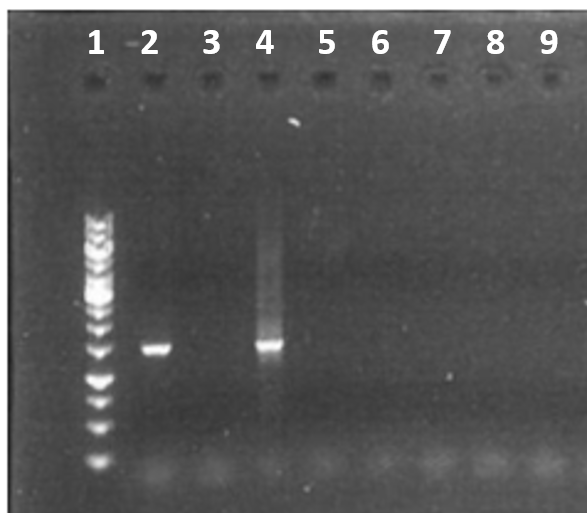


**Supplementary Figure 1.** Agarose gel electrophoresis of the PCR products of 16 rRNA amplification. 1: Ladder 1kB, 2: positive control Pseudomonas sp. DNA, 3: negative control PCR mix plus milliQ water without template DNA, 4: non-axenic *H. pluvialis* strain SAG192.8, 5-9: candidate axenic isolates of *H. pluvialis* strain SAG192.8.


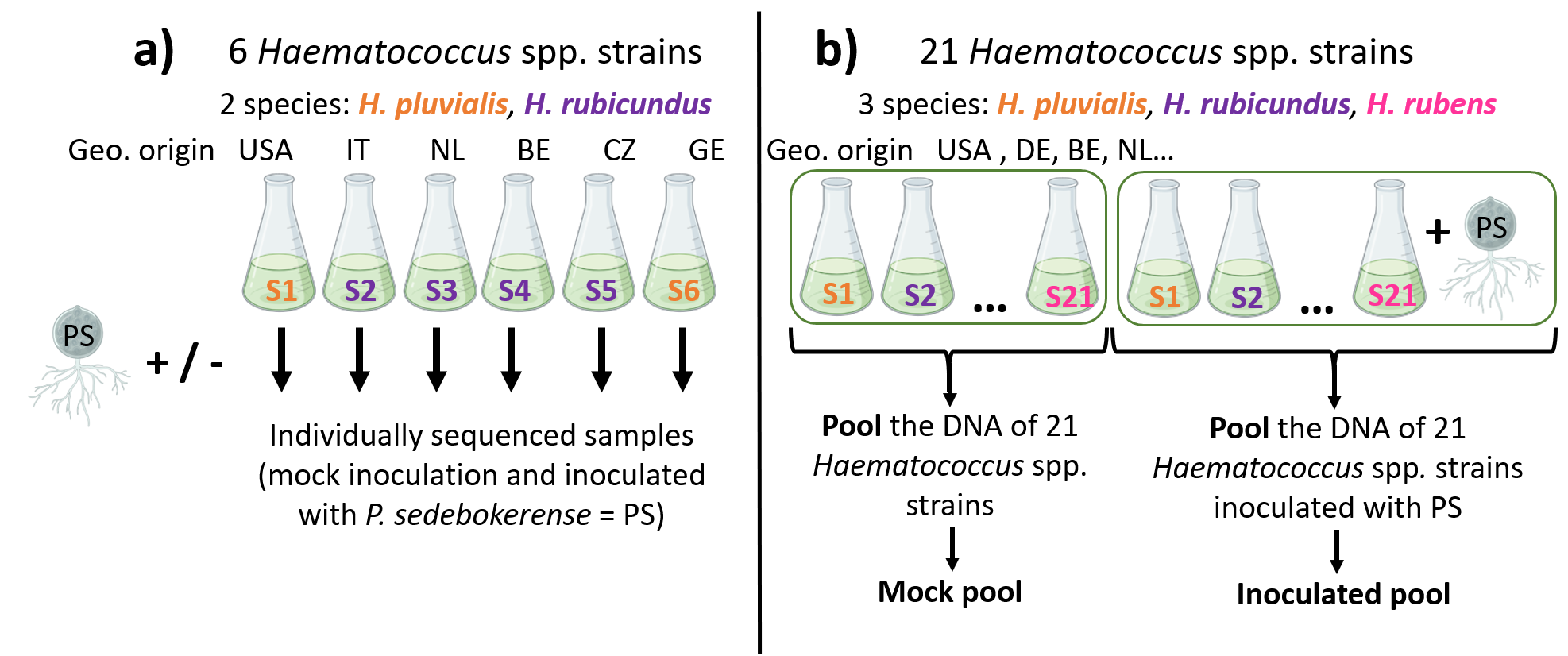


**Supplementary Figure 2.** Schematic representation of the metagenomic experimental design. S1= CCAP34/14, S2 = IT01_06, S3= NL02_08, S4= BE05_06, S5= CZ01_06, S6= SAG192.8. PS = P. *sedebokerense*.


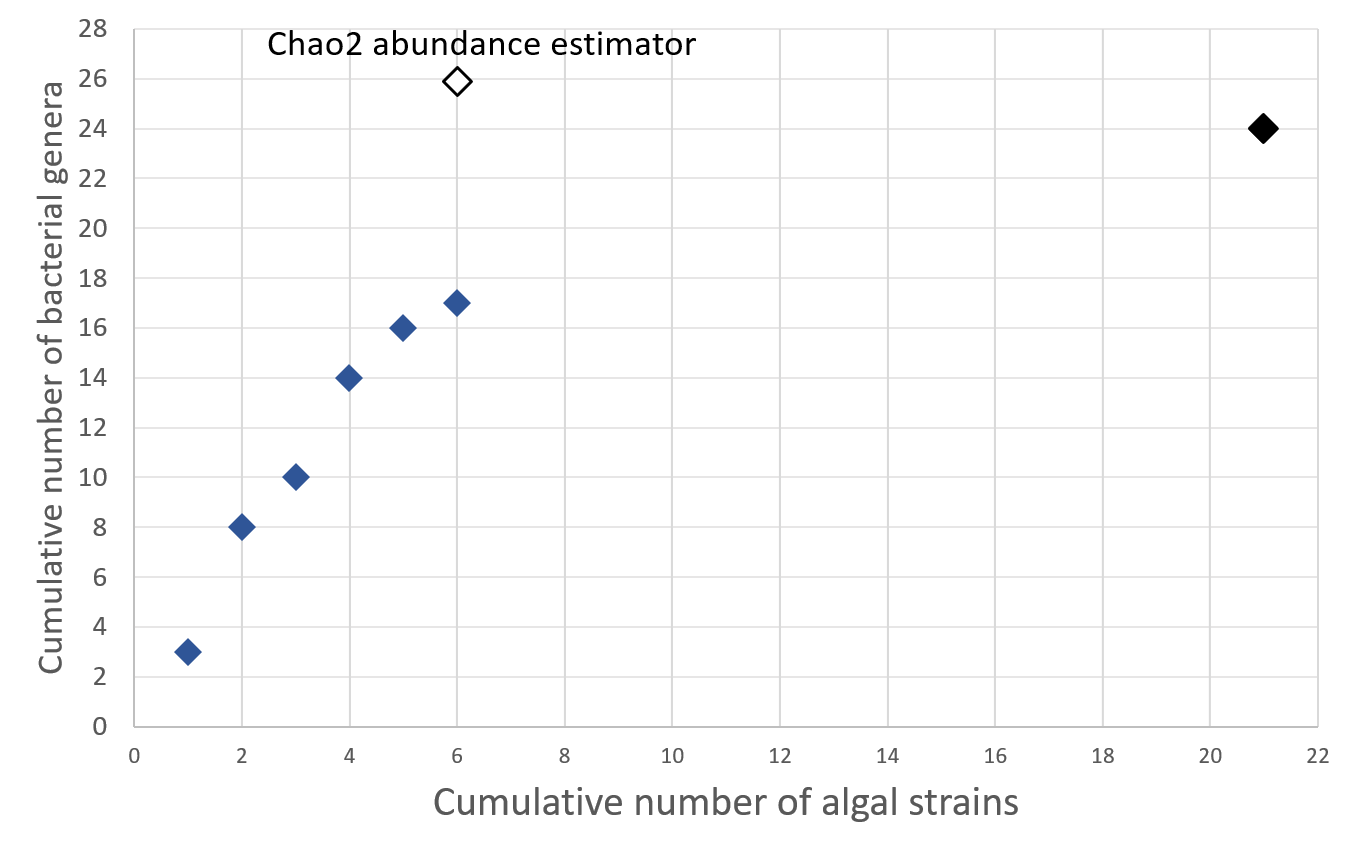


**Supplementary Figure 3.** Estimation of the overall microbiota diversity of laboratory-cultivated *Haematococcus*. Accumulation curve representing the non-redundant bacterial genera identified across the six individually sequenced *Haematococcus* strains (blue diamonds). The white diamond represents the total diversity estimated with the Chao2 index calculated on the six individually sequenced *Haematococcus* strains. The cumulative number of bacterial genera identified in the pooled sample is shown with a black diamond.


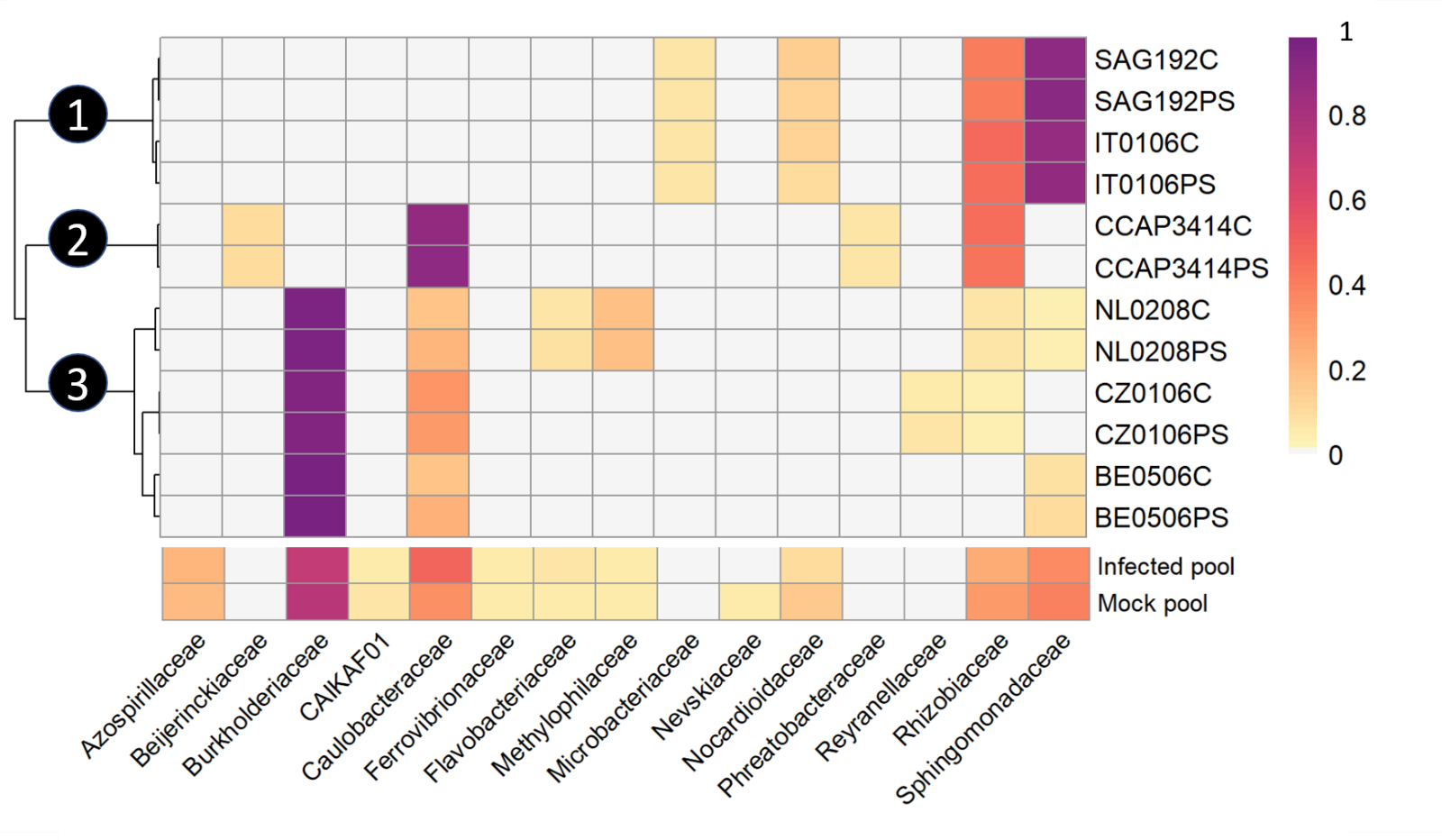


**Supplementary Figure 4.** Heatmap of the bacterial **families** identified by metagenomics across the *Haematococcus* strains when inoculated with the pathogen *P. sedebokerense* (PS) or in the control condition (C). The heatmap is based on bin abundance per sample normalized by the size of each library, *i.e.* the total number of reads in each sample, summed per family. The colour scale corresponds to the abundance matrix normalized using Hellinger transformation. Euclidean method for dissimilarity index calculation was used for clustering. Pooled samples were not included in the clustering. 1: Type 1 microbiota, 2: Type 2 microbiota, 3: Type 3 microbiota.


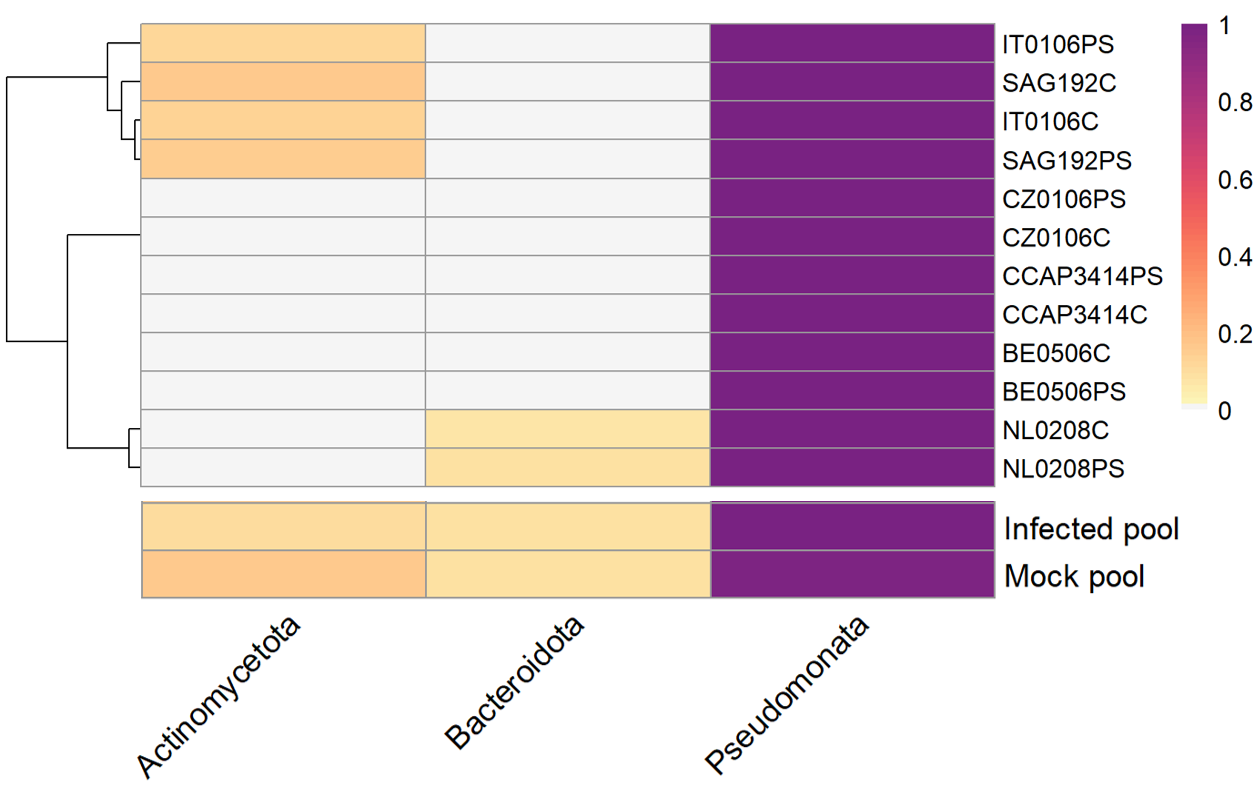


**Supplementary Figure 5.** Heatmap of the bacterial **phyla** identified by metagenomics across the *Haematococcus* strains when inoculated with the pathogen *P. sedebokerense* (PS) or in the control condition (C). The heatmap is based on bin abundance per sample normalized by the size of each library, *i.e.* the total number of reads in each sample, summed per phylum. The colour scale corresponds to the abundance matrix normalized using Hellinger transformation. Euclidean method for dissimilarity index calculation was used for clustering. Pooled samples were not included in the clustering.


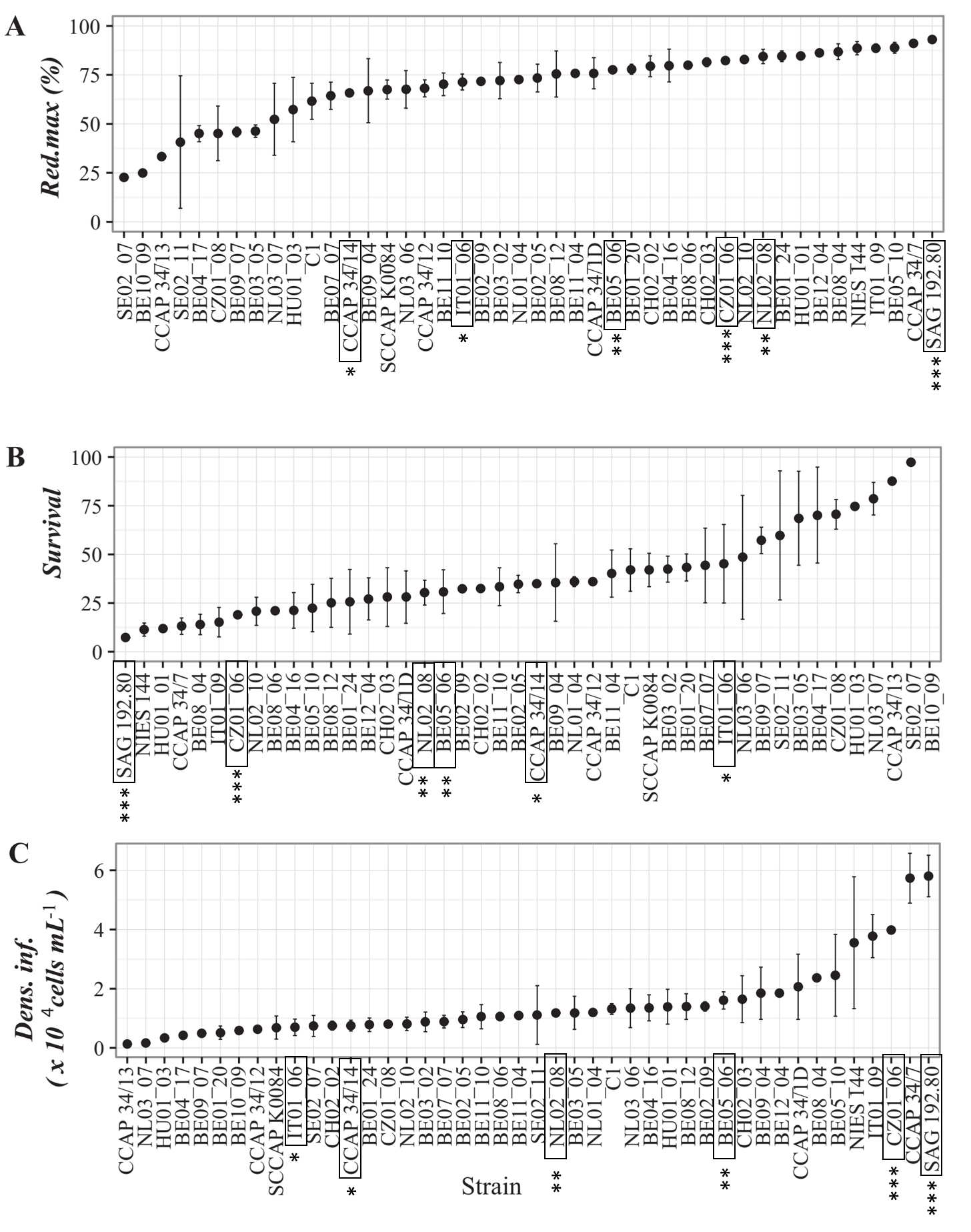


**Supplementary Figure 6.** Determination of *Haematococcus* susceptibility to *P.* *sedebokerense* by Allewaert et al., (2018). This figure was drawn from the original Figure 2 from (Allewaert et al., 2018): A: “Means ± SD (n=3) of the maximum percentage reduction of algal growth, Red. max. (%). B. Survival ratio of the algal cells and C. Density of infected *Haematococcus* cells (× 10^4^ cells mL^−1^) Dens. inf. of 44 *Haematococcus* strains. Strains are ordered according to increasing mean for each variable.” Classification into three susceptibility subtypes for the six strains used individually in this study. *: low susceptibility, **: intermediate susceptibility, ***: high susceptibility based on Allewaert et al., (2018).


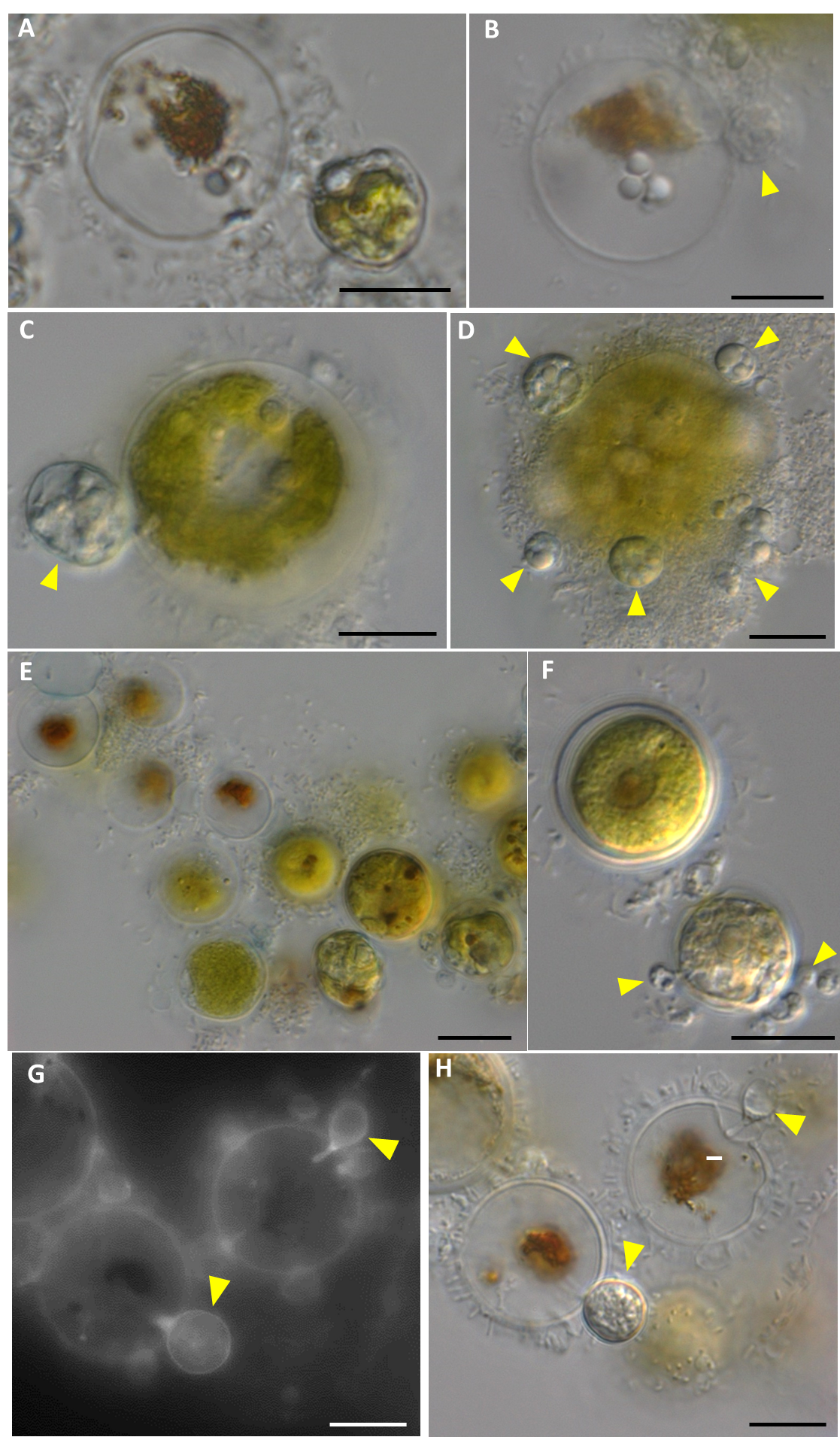


**Supplementary Figure 7.** Microscopy images of a selection of the *Haematococcus* cultures inoculated with *P. sedebokerense* for the metagenomics experiment representative of all strains used and showing visible signs of infection. *P. sedebokerense* cells are visible at the algal surface (yellow arrowheads). *Haematococcus* strains (A) NL03_06, (B) BE05_16, (C) Haemc1, (D) CH02_08, (E) NL02_10 and (F) BE05_06., (G) and (H) BE01_20, (G) calcofluor white staining highlights the presence of the fungal pathogen and the development of fungal rhizoids inside the infected algal cells. Scale bar 10 μm.


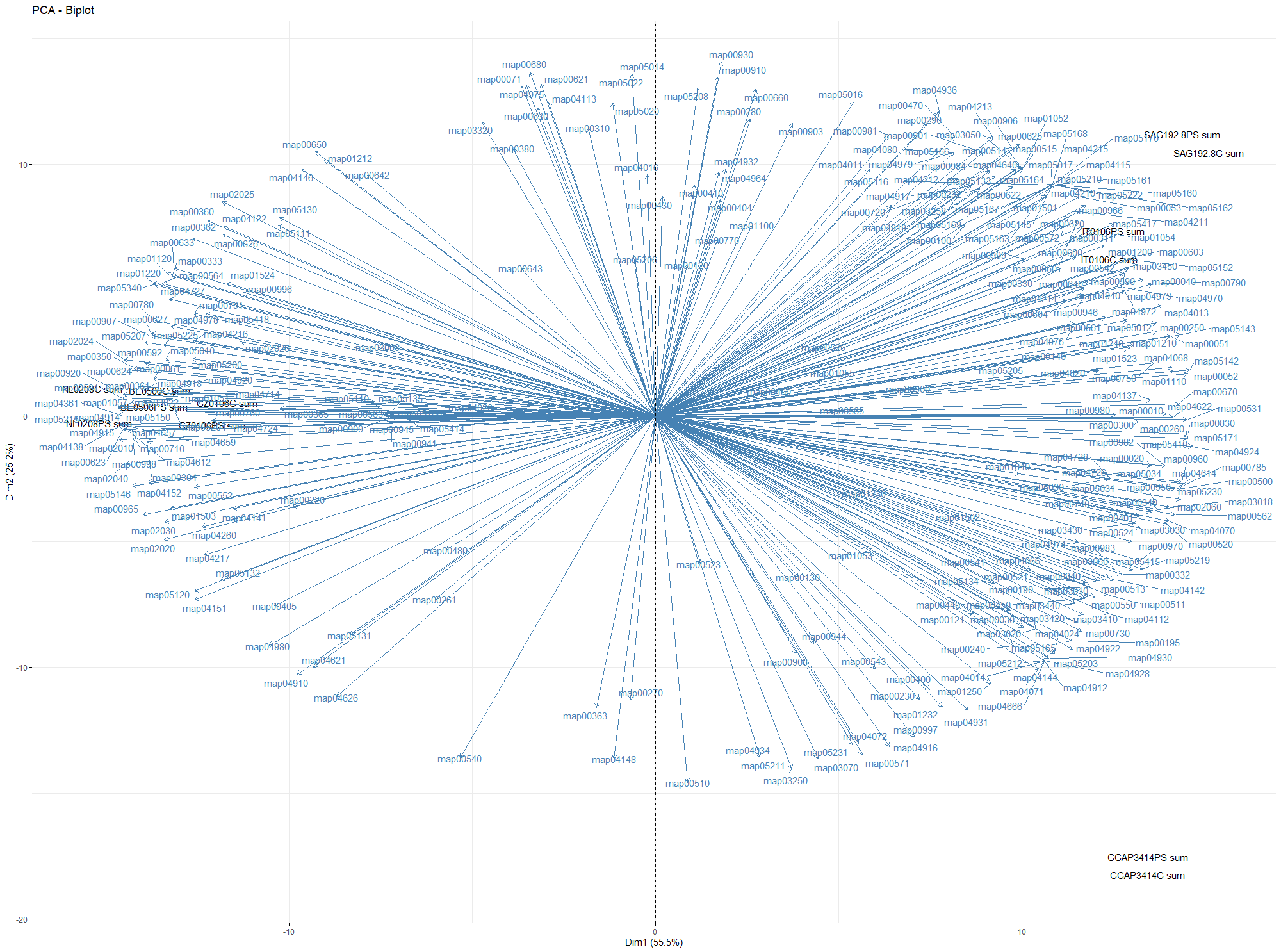


**Supplementary Figure 8.** Results of the Principal Component Analysis (PCA) based on the matrix with the number of reads mapping on annotated features (KOs), normalised to 1kB and per million read of annotated KOs within each library (sample) and then summed per KO for all MAGs within a library (sample), the KOs were then grouped in functional KEGG pathway (map). Maps as variables are shown. Projection on the 1^st^ and 2^nd^ components.

# References:

Allewaert, C.C., Hiegle, N., Strittmatter, M., de Blok, R., Guerra, T., Gachon, C.M.M., Vyverman, W., 2018. Life history determinants of the susceptibility of the blood alga Haematococcus to infection by Paraphysoderma sedebokerense (Blastocladiomycota). Algal Res. 31, 282–290. https://doi.org/10.1016/j.algal.2018.02.015

Blifernez-Klassen, O., Klassen, V., Wibberg, D., Cebeci, E., Henke, C., Rückert, C., Chaudhari, S., Rupp, O., Blom, J., Winkler, A., Al-Dilaimi, A., Goesmann, A., Sczyrba, A., Kalinowski, J., Bräutigam, A., Kruse, O., 2021. Phytoplankton consortia as a blueprint for mutually beneficial eukaryote-bacteria ecosystems based on the biocoenosis of Botryococcus consortia. Sci. Rep. 11, 1726. https://doi.org/10.1038/s41598-021-81082-1

Carney, L., Sorensen, K., 2015. Haematococcus pluvialis culture compositions.

Chekanov, K., Zaytseva, A., Mamedov, I., Solovchenko, A., Lobakova, E., 2021. The Dynamics of the Bacterial Community of the Photobioreactor-Cultivated Green Microalga Haematococcus lacustris during Stress-Induced Astaxanthin Accumulation. Biology 10, 115. https://doi.org/10.3390/biology10020115

Hoeger, A.-L., Griehl, C., Noll, M., 2021. Infection with intracellular parasite Amoeboaphelidium protococcarum induces shifts in associated bacterial communities in microalgae cultures. J. Appl. Phycol. 33, 2863–2873. https://doi.org/10.1007/s10811-021-02542-9

Kim, B.-H., Ramanan, R., Cho, D.-H., Oh, H.-M., Kim, H.-S., 2014. Role of Rhizobium, a plant growth promoting bacterium, in enhancing algal biomass through mutualistic interaction. Biomass Bioenergy 69, 95–105. https://doi.org/10.1016/j.biombioe.2014.07.015

Krohn-Molt, I., Alawi, M., Förstner, K.U., Wiegandt, A., Burkhardt, L., Indenbirken, D., Thieß, M., Grundhoff, A., Kehr, J., Tholey, A., Streit, W.R., 2017. Insights into Microalga and Bacteria Interactions of Selected Phycosphere Biofilms Using Metagenomic, Transcriptomic, and Proteomic Approaches. Front. Microbiol. 8, 1941. https://doi.org/10.3389/fmicb.2017.01941

Kublanovskaya, A., Solovchenko, A., Fedorenko, T., Chekanov, K., Lobakova, E., 2019. Natural Communities of Carotenogenic Chlorophyte Haematococcus lacustris and Bacteria from the White Sea Coastal Rock Ponds. Microb. Ecol. 79, 785–800. https://doi.org/10.1007/s00248-019-01437-0

Lee, C., Jeon, M.S., Kim, J.Y., Lee, S.H., Kim, D.G., Roh, S.W., Choi, Y.-E., 2019. Effects of an auxin-producing symbiotic bacterium on cell growth of the microalga Haematococcus pluvialis: Elevation of cell density and prolongation of exponential stage. Algal Res. 41, 101547. https://doi.org/10.1016/j.algal.2019.101547

Lee, S.-A., Kim, M., Esterhuizen, M., Le, V.V., Kang, M., Ko, S.-R., Oh, H.-M., Kim, Y.J., Ahn, C.-Y., 2022. An acceleration of carotenoid production and growth of Haematococcus lacustris induced by host-microbiota network interaction. Microbiol. Res. 262, 127097. https://doi.org/10.1016/j.micres.2022.127097

Li, Y., Chen, X., Wang, Q., Liu, Y., Li, J., Gong, Q., Gao, X., 2022. Diversity and dynamics of bacterial communities associated with Haematococcus pluvialis at different life stages. J. Appl. Phycol. 34, 1353–1361. https://doi.org/10.1007/s10811-022-02729-8

Ramanan, R., Kang, Z., Kim, B.-H., Cho, D.-H., Jin, L., Oh, H.-M., Kim, H.-S., 2015. Phycosphere bacterial diversity in green algae reveals an apparent similarity across habitats. Algal Res. 8, 140–144. https://doi.org/10.1016/j.algal.2015.02.003
